# Multi‐timepoint pattern analysis: Influence of personality and behavior on decoding context‐dependent brain connectivity dynamics

**DOI:** 10.1002/hbm.25732

**Published:** 2021-12-03

**Authors:** Saampras Ganesan, Jinglei Lv, Andrew Zalesky

**Affiliations:** ^1^ Melbourne Neuropsychiatry Centre, Department of Psychiatry, The University of Melbourne Melbourne Victoria Australia; ^2^ Department of Biomedical Engineering The University of Melbourne Melbourne Victoria Australia; ^3^ School of Biomedical Engineering University of Sydney Sydney New South Wales Australia; ^4^ Brain and Mind Centre University of Sydney Sydney New South Wales Australia

**Keywords:** behavior, brain network, dynamic functional connectivity, functional MRI, logistic regression, rest, task

## Abstract

Behavioral traits are rarely considered in task‐evoked functional magnetic resonance imaging (MRI) studies, yet these traits can affect how an individual engages with the task, and thus lead to heterogeneity in task‐evoked brain responses. We aimed to investigate whether interindividual variation in behavior associates with the accuracy of predicting task‐evoked changes in the dynamics of functional brain connectivity measured with functional MRI. We developed a novel method called multi‐timepoint pattern analysis (MTPA), in which binary logistic regression classifiers were trained to distinguish rest from each of 7 tasks (i.e., social cognition, working memory, language, relational, motor, gambling, emotion) based on functional connectivity dynamics measured in 1,000 healthy adults. We found that connectivity dynamics for multiple pairs of large‐scale networks enabled individual classification between task and rest with accuracies exceeding 70%, with the most discriminatory connections relatively unique to each task. Crucially, interindividual variation in classification accuracy significantly associated with several behavioral, cognition and task performance measures. Classification between task and rest was generally more accurate for individuals with higher intelligence and task performance. Additionally, for some of the tasks, classification accuracy improved with lower perceived stress, lower aggression, higher alertness, and greater endurance. We conclude that heterogeneous dynamic adaptations of functional brain networks to changing cognitive demands can be reliably captured as linearly separable patterns by MTPA. Future studies should account for interindividual variation in behavior when investigating context‐dependent dynamic functional connectivity.

## Graphical abstract

We developed a novel method called multi‐timepoint pattern analysis, in which binary logistic regression classifiers were trained to distinguish rest from each of 7 tasks (i.e., social cognition, working memory, language, relational, motor, gambling, emotion) based on functional connectivity dynamics measured in 1,000 healthy adults. We found that connectivity dynamics for multiple pairs of large‐scale networks enabled individual classification between task and rest with accuracies exceeding 70%, with the most discriminatory connections relatively unique to each task. Crucially, interindividual variation in classification accuracy significantly associated with several behavioral, cognition and task performance measures, such as perceived stress, aggression, alertness, and endurance. Future studies should account for interindividual variation in behavior when investigating context‐dependent dynamic functional connectivity.

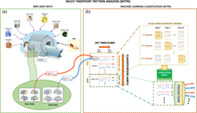

## 1 INTRODUCTION

Functional brain connectivity inferred from functional magnetic resonance imaging (fMRI) is dynamic in both space (Iraji, Miller, Adali, & Calhoun, 2020; Kottaram et al., 2018) and time (Hutchison, Womelsdorf, Allen, et al., 2013; Hutchison, Womelsdorf, Gati, et al., 2013; Preti, Bolton, & Van De Ville, 2017; Zalesky, Fornito, Cocchi, Gollo, & Breakspear, 2014). These dynamics underpin the rapid reorganization of functional brain networks in response to changing cognitive demands (Calhoun, Kiehl, & Pearlson, 2008; Cole, Bassett, Power, Braver, & Petersen, 2014; Linden et al., 1999) as well as endogenous fluctuations in network architecture during rest (Biswal, Van Kylen, & Hyde, 1997; Fox & Raichle, 2007; van den Heuvel & Hulshoff Pol, 2010). Endogenous and task‐evoked dynamics in functional connectivity are typically studied separately and salient differences in functional network dynamics between these two conditions remain unclear (Meer, Breakspear, Chang, Sonkusare, & Cocchi, 2020). While time‐averaged, or *static*, functional network architecture appears to be highly similar between rest and task, subtle task‐specific reorganization is evident within particular circuits (Cole et al., 2014) and recent studies suggest that complex topological properties such as network hubs (Bolt, Nomi, Rubinov, & Uddin, 2017) and modularity (DeSalvo, Douw, Takaya, Liu, & Stufflebeam, 2014; Tomasi, Wang, Wang, & Volkow, 2014) reorganize in response to changing cognitive demands. However, the extent to which time‐averaged analyses of functional brain networks can adequately characterize network reorganization is limited by the use of an inherently static representation of the brain.

Elucidating the temporal and spatial *dynamics* of functional brain network organization is likely to provide further insight into the salient differences in network architecture between rest and task engagement (Mill, Ito, & Cole, 2017). The dynamics of functional connectivity can be investigated at the scale of individual regions, individual connection between a pair of regions, or at the whole‐brain scale, in terms of putative brain states encompassing multiple regions. Dynamic brain states that represent temporally recurring functional network configurations have been mapped using approaches such as k‐means clustering (Allen et al., 2014) and hidden Markov models (Ou et al., 2015). At the scale of individual connections, connectivity dynamics have been quantified using measures such as standard deviation (SD) and coefficient of variation (Fong et al., 2019; Kucyi & Davis, 2014). Dynamics associated with individual regions have been mapped by estimating summary graph theory estimates, such as nodal efficiency, on successive time‐varying functional connectivity measures (Zalesky et al., 2014).

Classification of rest and task‐evoked functional connectivity dynamics is typically approached by identifying recurring patterns of functional connectivity that emerge during task performance. For instance, Zhang et al. (2013) applied a supervised dictionary learning technique to identify distinct spatial connectome patterns representing rest and task states. Individuals with overlapping rest and task connectome patterns were found to have poor compliance with the task paradigm. A study by Xie et al. (2019) used three different tasks and employed a combination of spatial independent components analysis, sliding window correlations and k‐means clustering to extract distinct spatial patterns of functional connectivity representing task and rest. For the working memory task, they found that individuals with misclassified network patterns also showed poor task performance. Similarly, distinct connectivity patterns distinguishing rest and task were identified by Denkova, Nomi, Uddin, and Jha (2019) and Cheng et al. (2018) using k‐means clustering of time‐resolved functional connectivity. Few studies have sought to differentiate task and rest using functional connectivity dynamics between individual pairs of regions. Moreover, unlike the state‐based approach which primarily utilizes spatial patterns of functional connectivity, edge‐based approaches have not considered the complete temporal patterns of connectivity and instead relied mostly on single variability measures. For instance, analyzing dynamics using sliding window functional connectivity, Elton and Gao (2015) found that task engagement leads to reduced connectivity variability compared to rest, and task performance significantly associates with this variability (Elton & Gao, 2015; Fong et al., 2019).

While some of these previous studies control for the impact of in‐ and out‐of‐scanner task performance on task‐evoked changes in functional connectivity dynamics, few studies have considered the impact of behavioral traits. Although methods have been recently developed to investigate the dynamic relationship between behavior and functional connectivity in the context of naturalistic stimulus, their focus is not on time‐resolved connectivity (Finn et al., 2020). It remains unknown whether temporal patterns of dynamic functional connectivity (DFC) can be used to differentiate task from rest, even if these factors are controlled. Interindividual variation in personality and behavior could potentially impact how an individual engages with a task, thus leading to heterogeneity in task‐evoked connectivity dynamics and impacting the ability to differentiate these dynamics from endogenous activity. Despite evidence of an association between the dynamic properties of functional connectivity and interindividual variation in emotion, personality, and cognitive performance (Bolton & Ville, 2020; Cohen, 2018; Gonzalez‐Castillo & Bandettini, 2018; Shi et al., 2018; Tobia et al., 2017; Viviano et al., 2017; Wu et al., 2019), it remains unclear whether these behavioral measures also associate with the extent to which rest and task can be differentiated based on connectivity dynamics.

To address this gap, we propose a novel multivariate and model‐free approach called multi‐timepoint pattern analysis (MTPA), which is distinct from established multi‐voxel pattern analysis (MVPA) methods. In MVPA, a supervised technique is applied to learn linearly or nonlinearly separable spatial patterns of voxel activity that differentiate experimental conditions (Norman et al., 2006). On the contrary, in MTPA, we employ supervised logistic regression to learn linearly separable temporal patterns in functional connectivity dynamics that can be used to predict whether an individual is engaged in a task or resting. The logistic regression classifier not only enables out‐of‐sample prediction but also provides a means to investigate the influence of behavioral measures on these predictions. To avoid arbitrary window size selection, retain maximum information from each paradigm as well as maximize the number of timepoint features available for MTPA, we estimated DFC using a high‐resolution frame‐wise method called flexible least squares (FLS; Kalaba & Tesfatsion, 1989; Liao, Wu, et al., 2014). Moreover, this is one of the first studies to investigate the dynamics of resting‐state and task‐evoked functional connectivity in a large sample of more than 1,000 healthy adults.

The aims of this study are twofold. First, we aimed to identify functional connections whose temporal dynamics could accurately classify between rest and each of seven tasks using the new MTPA method. We applied MTPA to unravel differences between connectivity associated with rest and task after mean task activation was removed. We hypothesized that relatively few connections would enable accurate out‐of‐sample classification, that these connections would be unique to each task and that their dynamic properties would yield higher classification accuracies than conventional time‐averaged functional connectivity. These hypotheses are supported by recent evidence suggesting that network reorganization between rest and different tasks can be characterized more accurately by functional connectivity dynamics (Cheng et al., 2018; Denkova et al., 2019; Fong et al., 2019; Xie et al., 2019; Zhang et al., 2013). Second, we aimed to investigate whether interindividual variation in behavioral measures, including emotion, alertness, and cognitive performance, explain interindividual variation in classification accuracies. Although it is well established that an individual's in‐scanner task performance is an accurate predictor of the ability to distinguish task from rest (Fong et al., 2019; Gonzalez‐Castillo & Bandettini, 2018; Xie et al., 2019; Zhang et al., 2013), we hypothesized that behavioral measures not directly related to the task would also influence classification accuracy. This hypothesis is motivated by mounting evidence suggesting that physiological states (i.e., drowsiness, etc.) and psychological traits (i.e., anxiety and stress, etc.) can impact functional connectivity (Denkova et al., 2010; Liégeois et al., 2019; Liston et al., 2009; Luettgau et al., 2018; Nair et al., 2020; Tobia et al., 2017). Our findings provide new insight into the functional connectivity dynamics associated with task engagement and draw attention to the importance of modeling behavioral characteristics in task fMRI experiments.

## 2 MATERIALS AND METHODS

### 2.1 Data and experimental design

Minimally preprocessed resting‐state and task‐evoked fMRI data were obtained from the Human Connectome Project (HCP). Data were acquired during rest (resting‐state fMRI) and seven tasks (task‐evoked fMRI) using a 3 T Connectome Skyra MRI scanner with TR = 720 ms; TE = 33.1 ms; flip angle = 52°; BW = 2,290 Hz/Px; in‐plane FOV = 208 × 180 mm; 72 slices; 2.0 mm isotropic voxels, with a multiband acceleration factor of 8. In this study, data acquired with left‐to‐right phase encoding was used for all analyses. The duration of acquisition varied between tasks, ranging between 2 and 5 min. For all seven tasks, minimally preprocessed data were available for about 1,000 healthy adults ranging in age between 22 and 37 years. Minimally preprocessed left‐to‐right phase‐encoded resting‐state fMRI data for the same individuals was also obtained. Spatial preprocessing of task and resting‐state fMRI data included gradient unwarping, motion correction, spatial distortion correction, field bias reduction, registration to structural T1‐weighted scans, nonlinear registration to MNI152 space, grand‐mean intensity normalization and brain masking. Experimental designs, data acquisition parameters, and preprocessing pipelines are described in detail elsewhere (Glasser et al., 2013; Smith et al., 2013). The seven tasks analyzed in this study were social cognition (shapes move socially or randomly); working memory (zero‐back and two‐back); language (story comprehension or math); relational processing (pattern relation or pattern matching); motor (move hands, feet, or tongue); emotion processing (match faces with expressions, or shapes); and gambling (guessing and winning/losing). All tasks followed a block design, where each block comprised one of multiple task conditions. Supplementary Table ST1 provides a brief summary of the task design and individuals included for each task.

We also performed some additional preprocessing steps on the minimally preprocessed data to account for head motion and physiological fluctuations. Specifically, we corrected for head motion by regressing out the variance associated with the frame‐wise displacement (FD) measure from both the task and resting‐state fMRI data of each individual. Effects due to physiological confounds prevalent in resting‐state fMRI data were removed by regressing out the variance associated with the average ventricular and white matter signals. To separate the effects of dynamic connectivity from first‐order time‐locked co‐activation (Laumann et al., 2017), we also regressed out the variance associated with the respective block designs of each task, prior to connectivity calculations. Specifically, this involved running general linear models (GLMs) for each individual with their respective FD and canonical HRF‐convolved task regressors, and estimating connectivity from the resulting residuals. Therefore, this allowed us to investigate the residual differences in the DFC between task and rest. Supplementary Analysis SA1 discusses the results of the same analysis without the above‐mentioned steps, that is, head motion correction, physiological correction, and mean task activation removal.

### 2.2 DFC estimation

We used the DynamicBC toolbox (Liao, Wu, et al., 2014) to estimate frame‐wise whole‐brain DFC. Frame‐wise DFC estimates time‐resolved functional connectivity at the temporal resolution of the fMRI acquisition (i.e., TR). DFC was computed using a time‐varying regression approach known as FLS (Kalaba & Tesfatsion, 1989; Liao, Wu, et al., 2014). FLS uses a state‐modeling based filtering approach and estimates a state (i.e., beta coefficient) for each individual time point by minimizing the errors associated with (a) discrepancies between the actual and estimated observation at each time point (measurement fit error), and (b) discrepancies due to incorrect specifications of the state transition equations (dynamic error). Both errors are characterized by ordinary least squares estimation. This is in contrary to static linear regression, where a single β coefficient is obtained by minimizing the residual fit error between two signals (Kalaba & Tesfatsion, 1989). The measurement fit error is given by

(1) $$\;r_{M}^{2}\left(\beta ,T\right)=\sum_{t=1}^{T}{\left(y\left(t\right)-x\left(t\right)\beta \left(t\right)\right)}^{2},$$

where *x*(*t*) and *y*(*t*) represent the values of two individual fMRI time‐series at time *t*, *T* is the total number of time points in the given time‐series, and *β* denotes the model fit coefficient vector. Dynamic error is given by

(2) $$r_{D}^{2}\left(\beta ,T\right)=\sum_{t=1}^{T-1}{\left(\beta \left(t+1\right)-\beta \left(t\right)\right)}^{2},$$

The errors in Equations (1) and (2) are combined into a single cost‐incompatibility function weighted by a Lagrange multiplier *μ*, thus enabling multicriteria optimization. This function is minimized to estimate the *β* coefficient sequence, formally:

(3) $$\;C\left(\beta ,\mu ,T\right)=\mu .r_{D}^{2}\left(\beta ,T\right)+r_{M}^{2}\left(\beta ,T\right),$$

where *μ* denotes the weighting parameter (Lagrange multiplier) between the measurement fit error, *r*_*M*_^2^, and the dynamic error, *r*_*D*_^2^. The incompatibility cost function was minimized with ordinary least squares estimation, permitting the *β*(*t*) coefficients to vary over time. Therefore, each FLS estimate (i.e., *β*(*t*) coefficient) demonstrates how the state vector could have evolved over time in a manner that minimizes the cost incompatibility function thereby maximizing the trueness of the priors defined in Equations (1) and (2). The weighting parameter *μ* arbitrates a trade‐off between erratic solutions with large dynamic errors (i.e., small *μ*) and solutions that tend toward the static linear regression solution (i.e., large *μ*) (Kalaba & Tesfatsion, 1989). Here, we used the default setting of *μ* = 100 since this produced optimal variation in the *β* sequence, as shown by Liao, Wu, et al. (2014). Note that this default setting is independent of the temporal resolution of fMRI acquisition (i.e., TR) and none of the equations governing FLS depend on the interval between time points. Furthermore, FLS does not make any assumptions about the probability distribution of the data. It is a data‐driven and distribution‐free method of estimating frame‐wise DFC, given an optimal TR‐independent *μ* of 100 (Kalaba & Tesfatsion, 1989; Liao, Wu, et al., 2014). As a result, FLS is not limited by requirements of prior window length specifications associated with the commonly used sliding‐window DFC, where an optimal choice of window length is arbitrary (Hutchison, Womelsdorf, Allen, et al., 2013; Hutchison, Womelsdorf, Gati, et al., 2013; Preti et al., 2017; Zhuang et al., 2020).

Frame‐wise DFC was computed separately for each individual and each task‐evoked and resting‐state fMRI acquisition, after the additional preprocessing steps as described above (see Supplementary Analysis SA1 for details of the analysis repeated with the minimally preprocessed version, without the additional steps). To this end, regionally averaged fMRI time‐series were determined for each of 150 regions comprising an established volumetric parcellation atlas of the cortex (Craddock et al., 2012). For each individual, frame‐wise DFC was computed for each of the 11,175 distinct pairs of regions using the FLS method described above. This produced a 150 × 150 × *T* matrix of frame‐wise DFC estimates (i.e., *β*(*t*) coefficients) for each individual and each task‐evoked and resting‐state fMRI acquisition, where *T* is the total number of time points comprising the acquisition. To match the time length of resting‐state data with that of task data, the time series of resting‐state DFC estimates were trimmed to only include the first *T* frames. MTPA was then performed on the resulting DFC estimates.

### 2.3 Static functional connectivity estimation

Time‐averaged functional connectivity was computed between each pair of regions using the Pearson correlation coefficient. Correlation coefficients were determined for the same regionally averaged, trimmed and additionally preprocessed fMRI time‐series described above. This yielded a 150 × 150 matrix of static functional connectivity estimates for each of the seven tasks and rest per individual.

### 2.4 Multi‐timepoint pattern analysis

Linear logistic regression classifiers were trained to distinguish rest from each of the seven tasks based on either time‐resolved (i.e., dynamic) or time‐averaged (i.e., static) functional connectivity. A separate classifier was trained for each pair of regions, yielding 11,175 independent classifiers for each of the seven tasks. Logistic regression is computationally efficient and enables individual‐level prediction. We implemented L2‐regularized linear logistic regression using the LIBLINEAR tool (Fan et al., 2008), as formulated in Supplementary Methods SM1. Note that the classification model outputs a probability estimate value for each prediction. This represents the probability of a time course of DFC *β*(*t*) coefficients belonging to one label or the other. For probability estimates ≥0.5, the assigned label is 1 (task) and otherwise 0 (rest).

#### 2.4.1 Dynamic functional connectivity

**FIGURE 1:**
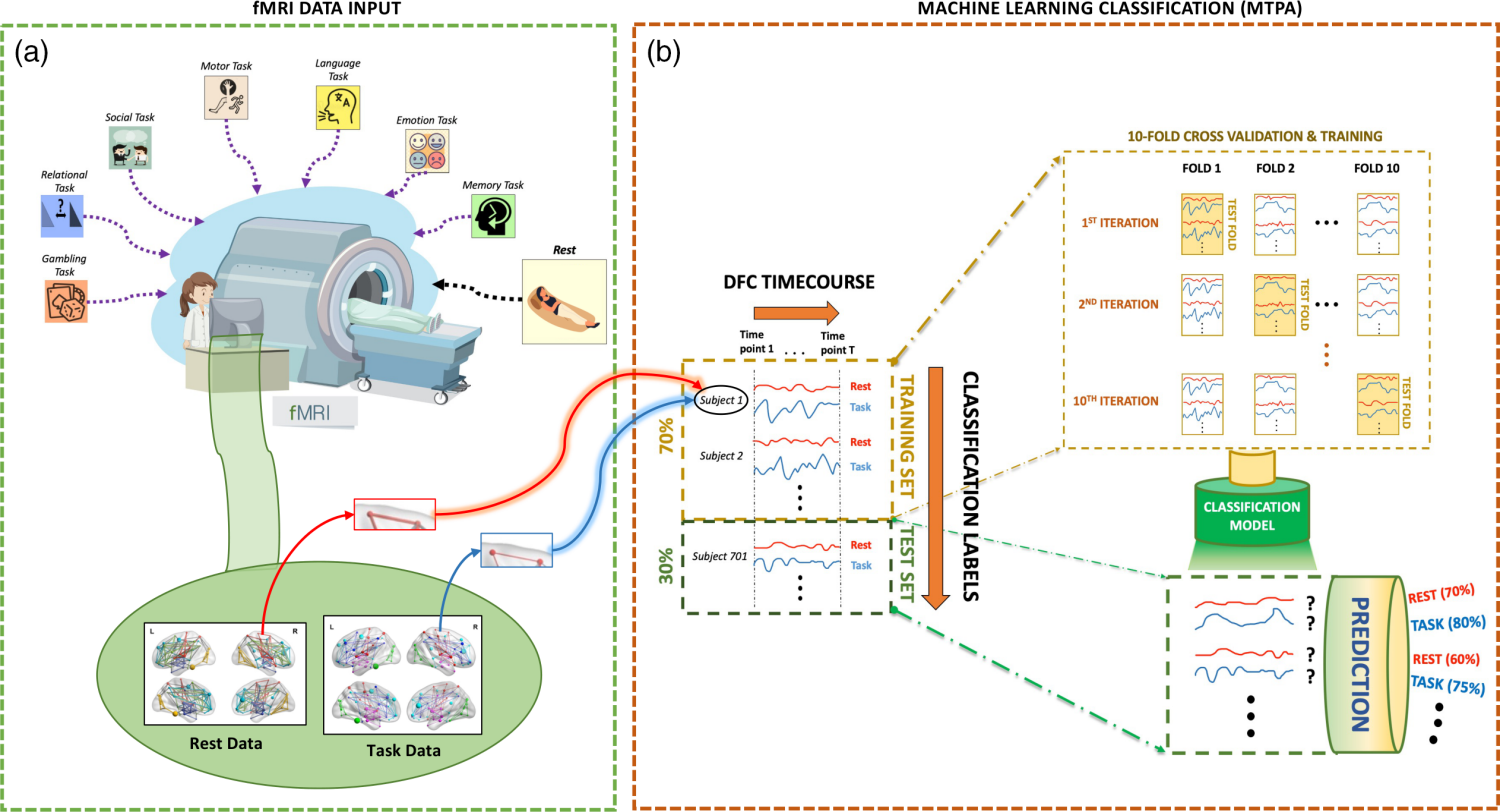
Schematic of the overall workflow for classifying task and rest using multi‐timepoint pattern analysis applied to dynamic functional connectivity. (a) Resting‐state and task‐evoked functional magnetic resonance imaging (MRI) data were analyzed for 7 tasks in more than 1,000 individuals. Dynamic functional connectivity was estimated between pairs of regions comprising an established parcellation atlas for each task. (b) For each pair of regions, a linear logistic regression model was trained to classify each of the seven tasks from rest based on dynamic functional connectivity. Training was performed using 10‐fold cross‐validation on 70% of the participants and classification accuracy was evaluated using an independent test set comprising 30% of the participants. Note that each prediction is accompanied by a corresponding probability estimate, represented by percentages here

The 150 × 150 × *T* DFC matrices comprising all unique region pairs from each individual were rearranged into separate two‐dimensional DFC matrices per region pair (one region pair × all individuals). Training and test sets were formed by randomly assigning 70% of all individuals to the training set and the remaining 30% to the test set. A separate model was trained for each of the 11,175 pairs of regions. The feature space comprised *T* frame‐wise DFC estimates (i.e., *β*(*t*) coefficients). We thus trained a total of 78,225 (11,175 pairs × 7 tasks) binary linear classification models. Subsequently, 10‐fold cross‐validation was performed on the training set to avoid model overfitting and provide a robust estimate of the accuracy with which rest could be distinguished from task. The accuracy of the model was further assessed by applying the trained models to individuals comprising the test set and generating out‐of‐sample predictions. Figure 1 provides a schematic of the overall workflow.

Each pair of regions for each of the seven tasks was associated with an out‐of‐sample estimate of prediction accuracy reflecting the accuracy with which the task could be differentiated from rest intrinsically. The prediction accuracies were rearranged to form a 150 × 150 symmetric matrix for each task. Using the large‐scale network atlas developed by Yeo et al. (2011), the matrix elements were further grouped into 17 canonical networks. For regions that could not be unequivocally assigned to a network, we demarcated three additional network categories; namely, “subcortical,” “cerebellum,” and “hippocampus/para hippocampus,” based on labels comprising the Harvard‐Oxford anatomical atlas distributed with FSL (http://www.fmrib.ox.ac.uk/fsl/). To reduce dimensionality and improve interpretability, we then averaged the prediction accuracies of pairs of regions within each network boundary to enable interpretation at the scale of broad networks. This resulted in a 20 × 20 matrix of prediction accuracies for each task.

Henceforth, we use *connections* to refer to pairs of regions comprising the parcellation atlas and *internetwork connections* to refer to connections between the 20 large‐scale networks. *Intranetwork connections* refer to connections within a given large‐scale network.

#### 2.4.2 Static functional connectivity

Classification of task and rest was also undertaken using conventional time‐averaged functional connectivity. Analogous to the classifiers trained using DFC, 11,175 linear logistic regression classifiers were trained to classify between task and rest from a one‐dimensional feature space comprising the static functional connectivity for each pair of regions. Thereafter, using the same approaches as above, a 20 × 20 matrix of prediction accuracies was obtained for each task, enabling explicit comparison with the corresponding matrices for DFC.

### 2.5 Associations with behavior and other measures

We investigated 192 behavioral, psychiatric, and biological measures available in the HCP. The behavioral measures evaluated include in‐scanner task performance, cognition, emotion, alertness, personality, motor ability, and alcohol use. The biological measures include age, sex, zygosity, and brain structure. We also investigated family history of psychiatric illness. To explore the role of emotion and personality on task‐rest discriminability, we included all emotion measures and summary scores associated with the top five personality types, that is, neuroticism, agreeableness, openness, conscientiousness, and extraversion. Since the level of alertness could influence task engagement, we also included all the sleep quality scores, while excluding most of the specific questionnaire responses. In addition, measures indicating amount of alcohol consumption over 7 days were also included. Finally, to explore the possibility of influence by brain structure in task modulation of brain dynamics, we included measures of average gray matter and white matter volumes. Supplementary Table ST2 lists the full set of measures that were investigated.

We used the Pearson partial correlation coefficient to test for associations between prediction accuracies (task vs. rest) and interindividual variation in each of the above 192 measures, partialling out the effect of age and sex. We corrected for multiple comparisons in each task category across the 192 tests using Bonferroni correction (*p* < .05/192). Significant associations with an effect size of *r* > |.1| were reported. Supplementary analyses were undertaken in which key measures of cognitive performance and in‐scanner task performance were also partialled out, in addition to age and sex (see Supplementary Methods SM2).

## 3 RESULTS

### 3.1 Classifying task and rest using functional connectivity dynamics

We investigated the extent to which the dynamics of functional connectivity differed between rest and seven tasks after regressing out their respective mean task activation: social cognition, working memory, language, relational processing, motor, emotional processing, and gambling. To this end, linear logistic regression classification was used to classify and predict between each of the seven tasks and rest, based on functional connectivity dynamics. An independent classifier was trained for each of 11,175 distinct connections using a 10‐fold cross‐validation procedure. Subsequently, the dimensionality was reduced by summarizing these connections into internetwork and intranetwork connections from 20 large‐scale canonical networks (Yeo et al. 2011), enabling meaningful network level interpretation for each task.

Prediction accuracies varied markedly between tasks and connections. We considered accuracies exceeding 70% for further analysis. While this threshold is somewhat arbitrary, we found that it enabled a reasonable balance between sensitivity and specificity. Using a lower threshold of 60% provided low specificity with more than 51% of the network connections exceeding the threshold on average across all seven tasks, whereas a higher threshold of 80% provided low sensitivity with fewer than 0.3% of connections exceeding the threshold on average. Moreover, a similar trend was observed for data that were not additionally preprocessed through motion and physiological corrections or removal of mean task activation (see Supplementary Analysis SA1). All accuracies reported here were estimated out of sample. However, the classification accuracies estimated in the training set using 10‐fold cross‐validation were comparable, indicating successful pattern classification without model overfitting. The initial parcellation into 150 distinct volumetric regions from the Craddock atlas was performed to enable classification on data derived from functionally homogenous brain regions with minimal loss of information due to averaging (Craddock et al., 2012). Note that 2 out of the 150 regions largely overlapped with the brain stem and hence were removed from analysis.

**FIGURE 2:**
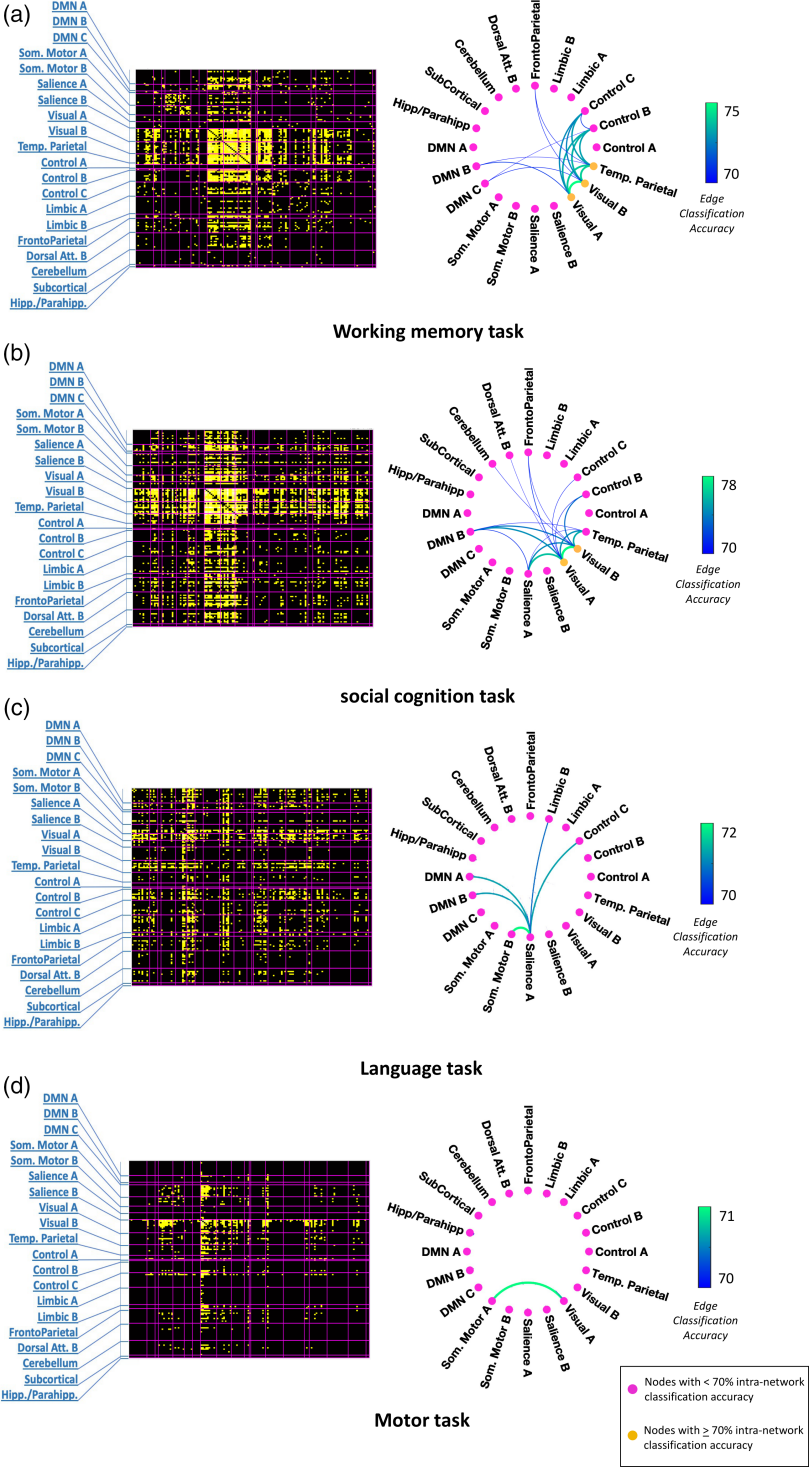
Accuracy of classifying between tasks and rest using dynamic functional connectivity. Left panel—Matrices of prediction accuracies (148 × 148) prior to downsampling to canonical networks for (a) working memory task, (b) social cognition task, (c) language task, and (d) motor task. Regions are delineated based on the Craddock volumetric atlas, with two regions of the brain stem removed. They are grouped according to canonical networks demarcated by magenta boundaries and indicated by labels. Each yellow square within a matrix represents a region‐to‐region connection whose prediction accuracy exceeded the set threshold. Right panel—Circular graph representations (using toolbox from https://github.com/paul‐kassebaum‐mathworks/circularGraph) of internetwork and intranetwork connections yielding average prediction accuracies exceeding the predefined threshold for four tasks, that is, (a) working memory task, (b) social cognition task, (c) language task, and (d) motor task. Each node represents an intranetwork connection while each edge represents an internetwork connection. The edge weights and edge colors (indicated by color bars) represent out‐of‐sample classification accuracy. Yellow nodes represent intranetwork connections whose dynamics enable prediction with accuracy exceeding 70%, while pink nodes represent intranetwork connections whose dynamics enable weaker prediction at below‐threshold accuracies

The left panel of Figure 2 shows the 148 × 148 matrices of out‐of‐sample classification accuracies prior to summarizing (i.e., downsampling) in terms of the 20 established canonical networks, while the right panel of Figure 2 shows circular graph representations of the summarized 20 × 20 matrix of averaged out‐of‐sample classification accuracies for each task with at least one network connection exceeding the threshold, that is, (a) working memory, (b) social cognition, (c) language, and (d) motor tasks. The accuracy matrices of the remaining tasks are shown in Supplementary Figure SF5. Each network connection in isolation was capable of an accuracy exceeding 70% on average. Possible interactions between multiple network connections were not considered.

Although the other three tasks, that is, relational, gambling and emotion, did not exceed the threshold when summarized into large‐scale brain networks, significant connections were evident at the scale of specific pairs of regions. For instance, in the relational task, the lateral occipital complex (LOC) showed widespread connections with the other visual occipital regions as well as the frontal pole and gyri. However, few connections were declared significant for the gambling and emotion tasks. For information about the region‐to‐region connections with the largest effect sizes for the remaining tasks, refer to Supplementary Results SR1, and Supplementary Figures SF2 and SF3.

When summarized into large‐scale networks, significant connections were found for four tasks. As shown in Figure 2, some networks were observed across multiple tasks. Internetwork connections associated with the visual subnetworks (A&B) were prominent in all the tasks except the language task, which was administered auditorily. The DMN subnetwork B, mainly comprising the superior and middle temporal gyri, also participated in three of the tasks (working memory, social cognition, and language) given its functional versatility in high‐level cognition and self‐referential processing (Gallagher & Frith, 2003; Howard et al., 2000; Schurz et al., 2014). Connectivity dynamics associated with the control subnetworks (B&C) that subserve executive processing were altered in all the three tasks requiring high‐level cognitive engagement, that is, working memory, social cognition, and language.

Some of the observed network connections were uniquely associated with specific tasks, consistent with the cognitive demands of the task. The dynamics of connectivity between the frontoparietal network and visual subnetworks were modulated in tasks necessitating high‐level visual attention and memory processes, that is, working memory (70.4% accuracy with visual B) and social cognition (71.3% accuracy with visual A and 70.3% accuracy with visual B). Dynamic connectivity between salience subnetwork A, dominated by insular regions, and DMN subnetworks was substantially altered in tasks that specifically involved high‐level emotional and socio‐cognitive processing, that is, social cognition (71.2% accuracy with DMN B) and language tasks (71% accuracy with DMN A and B). The dynamic coupling of temporoparietal network, associated with theory‐of‐mind processing and object recognition, with the visual and DMN subnetworks was distinct in both working memory (70.2% accuracy with DMN B, 73.8% with visual A and 73.9% with visual B) and social cognition tasks (70.5% accuracy with DMN B, 74.3% with visual A and 75.1% with visual B), compared to rest.

In the working memory task, the temporoparietal network, predominantly comprising the LOC, showed widespread task‐induced alterations in connectivity dynamics. Specifically, the dynamics of functional connectivity with itself (71.6% intranetwork accuracy), as well as with the visual, DMN, control (73% accuracy with control B, 72.9% with control C) and frontoparietal (70.4% accuracy) networks were distinct from those during rest. In the social cognition task, both the subnetwork B of DMN and the temporoparietal network showed dynamic alterations in their connectivity with each other, as well as with the visual subnetworks (74.3% DMN B—visual A, 73.6% DMN B—visual B) and salience subnetwork A (70.2% temporoparietal—salience A). On the other hand, alterations of DFC in the language task were only evident in the salience subnetwork A, through its connections with the DMN subnetworks A&B, somatomotor subnetwork B (71.6% accuracy), control subnetwork C (71% accuracy), and limbic subnetwork B (70.7% accuracy). In the cognitively less demanding motor task, only the connectivity between the somatomotor subnetwork A, dominated by supplementary motor areas, and the low‐level visual processing subnetwork A (70.6% accuracy) exhibited altered dynamics compared to rest.

**FIGURE 3:**
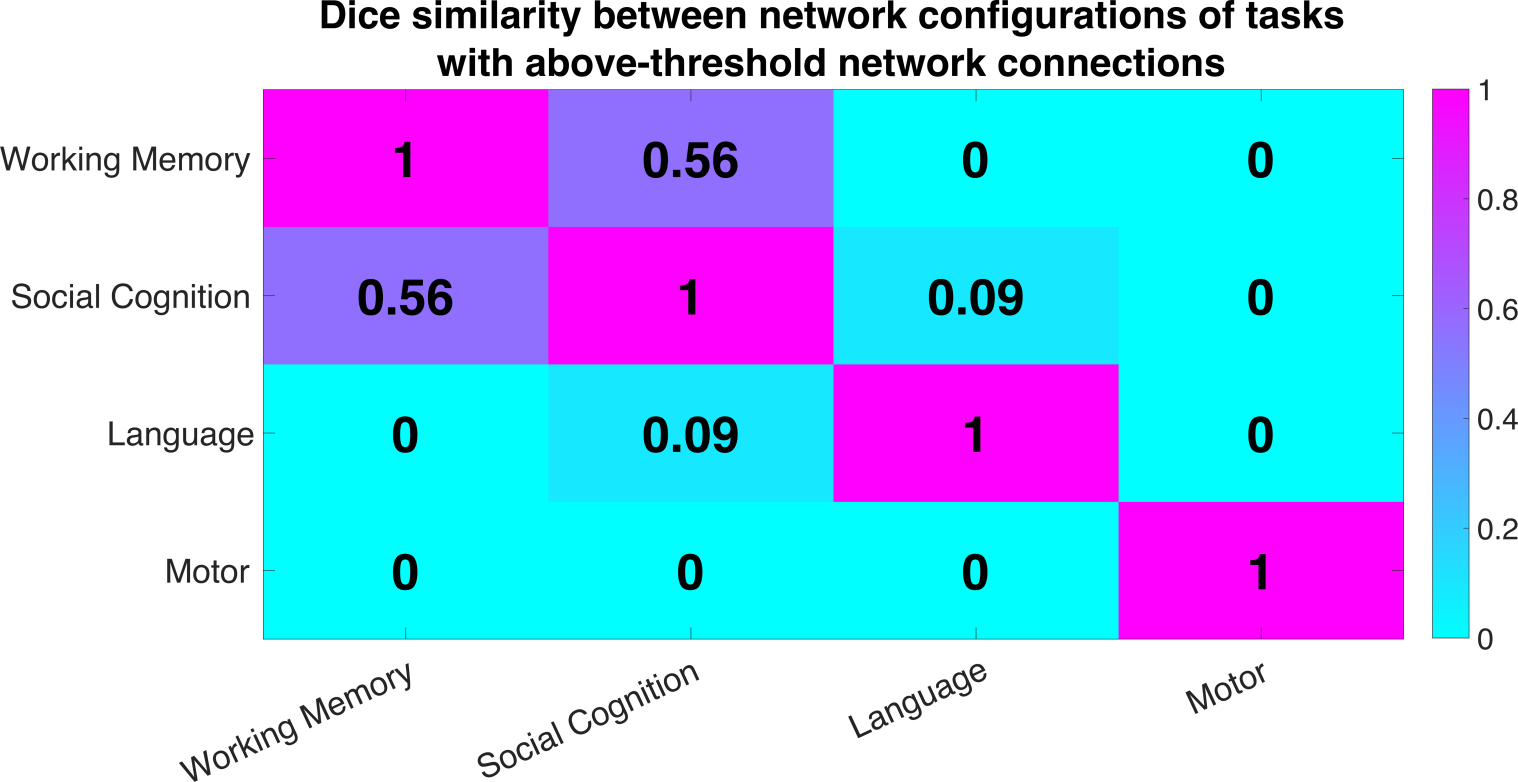
Similarity between network configurations enabling classification between task and rest using DFC. Similarity was assessed using the Dice similarity index computed between every pair of thresholded 20 × 20 network matrices of prediction accuracies. Note that the other three tasks, that is, relational, emotion, and gambling, were not included in this analysis as they did not produce any network connections that exceeded the prediction accuracy threshold

To quantitatively analyze the similarity between the network configurations showing task‐altered DFC in each of the four tasks, we calculated the dice similarity coefficient between the thresholded network‐level matrices (above 70% accuracy) of every pair of tasks (Figure 3). A dice index of 0 indicates no overlapping connections between two tasks, which was observed for all tasks with the cognitively less demanding motor task. Similarly, the network configuration of the auditorily presented language task hardly shared any similarity with the visually presented social cognition (dice index = 0.09) and working memory (dice index = 0) tasks, despite comparable cognitive requirements. On the other hand, there was a substantial overlap between the network configurations of working memory and social cognition tasks (dice index = 0.56), both of which involved higher‐order cognitive and memory processing while simultaneously attending to objects presented on a screen.

### 3.2 Classifying task and rest using static functional connectivity

**FIGURE 4:**
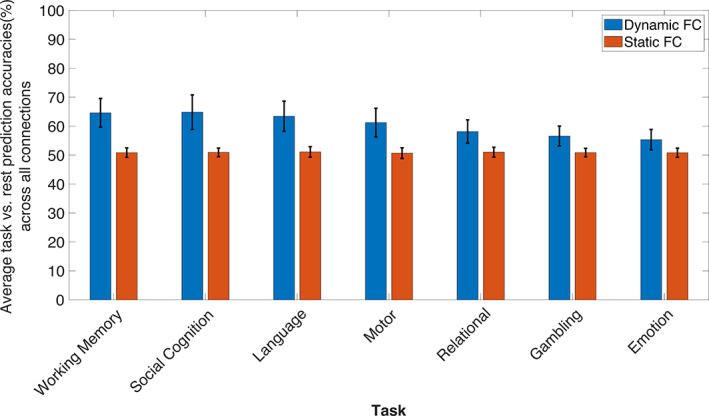
Average accuracy of discriminating between task and rest based on time‐averaged (orange) and dynamic (blue) functional connectivity computed from all the region‐to‐region connections. The mean out‐of‐sample prediction accuracy is shown for each of seven tasks. The standard deviation (SD*)* across connections is indicated by the vertical line along the top of each bar

To evaluate and compare the discriminability between task and rest enabled by dynamic and static functional connectivity measures, we performed MTPA on each static functional connection and followed the same approach as DFC (see Sections 2.4.1 and 2.4.2). The 148 × 148 matrices of prediction accuracies of all seven tasks prior to summarizing (i.e., downsampling) in terms of the 20 established canonical networks can be found in Supplementary Figure SF6. Network connections with a prediction accuracy exceeding 70% were not found for any of the tasks in the case of static functional connectivity. Furthermore, we also compared the absolute mean prediction accuracies obtained from dynamic and static functional connectivity by averaging across the whole 20 × 20 matrix of each task (Figure 4). We found that the average discrimination by static functional connectivity was comparable to chance level (50%) in every task. Additionally, the distribution of accuracies was largely centered around 50% (see Supplementary Figure SF7) for every task, thus indicating that static functional connectivity is not substantially different between task and rest, in the context of MTPA. This is consistent with previous studies that demonstrate only subtle differences between resting‐state and task‐based functional connectivity, despite intact task activation effects (Cole et al., 2014; Fair et al., 2007).

### 3.3 Impact of behavior on classifying task and rest using functional connectivity dynamics

Having found that task and rest can be accurately classified for four of the seven tasks based on dynamic connectivity, we next sought to investigate whether prediction accuracy was impacted by behavior and other individual‐specific measures. To this end, we computed the task identifiability measure for each individual and each task by averaging individual prediction probability estimates across all the region‐to‐region connections included in the thresholded network configuration. From the HCP dataset, we then selected 192 measures belonging to broad categories such as in‐scanner task performance of the four tasks, cognition, emotion, personality, and so forth. The Pearson partial correlation coefficient was used to test whether interindividual variation in each measure associated with classification performance, while controlling for the effects of age and sex. This was tested independently for each task and each measure. The Bonferroni correction was applied to correct for multiple comparisons across the 192 tests for each task.

**FIGURE 5:**
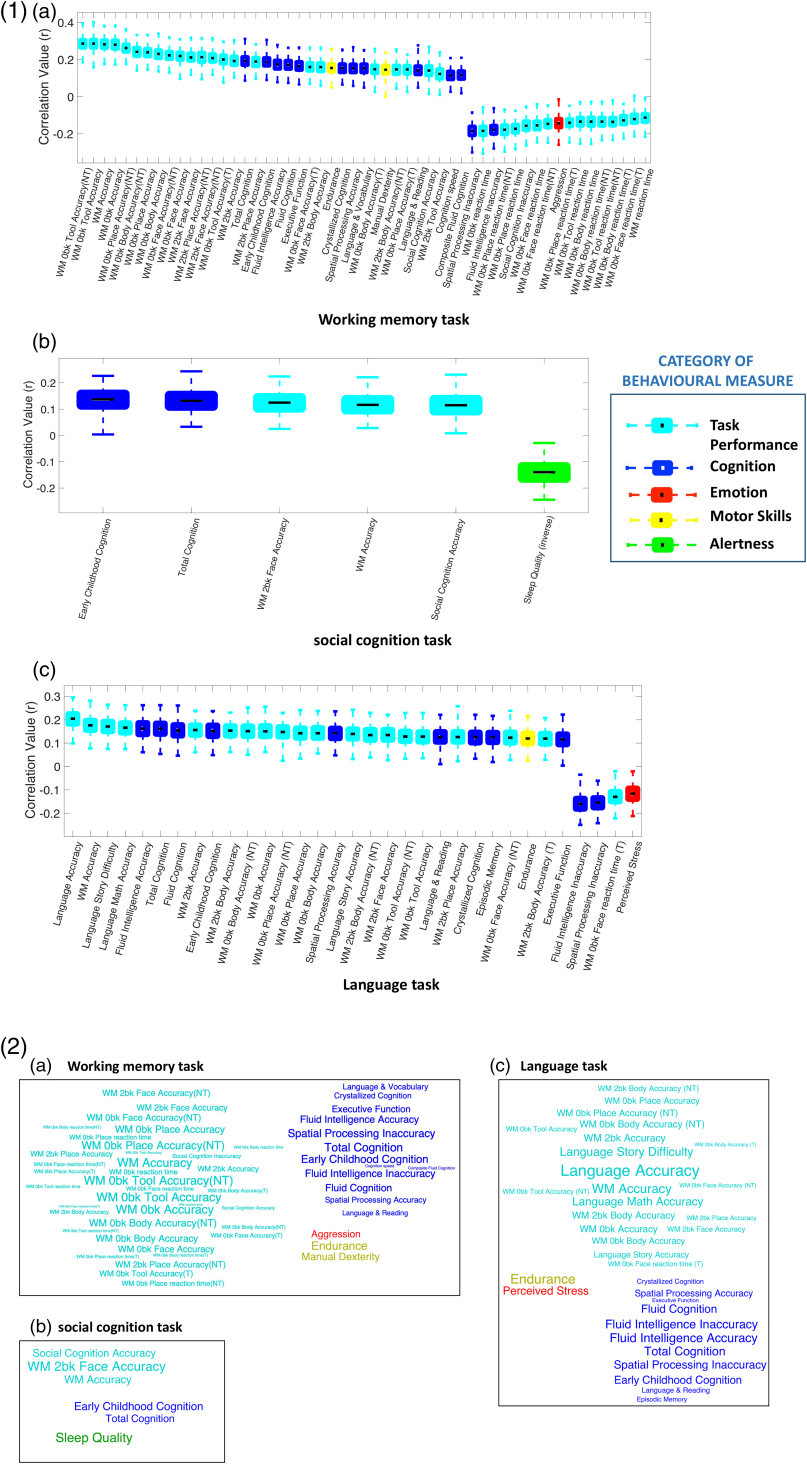
Qualitative and quantitative representations of the significant associations between behavioral measures and task identifiability measures. 1) Boxplots showing statistically significant correlations (*p* < .05, |*r*| > 0.1, Bonferroni corrected), with 75% confidence interval, between individual task identifiability measures and behavioral measures from (a) working memory task, (b) social cognition task, and (c) language task, after controlling for the influence of age and gender. 2) The word clouds at the bottom provide a qualitative representation of the correlations for (a) working memory task, (b) social cognition task, and (c) language task. The size of the word represents correlation strength, and the color represents behavioral category. Note that the font color in the word clouds matches the category colors defined for the boxplots. NT, nontarget trials; T, target trials; WM—working memory

For three of the tasks, that is, working memory, social cognition, and language, we found a significant relationship between the task identifiability measure and several behavioral measures associated with five distinct behavioral categories, namely in‐scanner task performance, cognition, emotion, alertness, and motor ability. Figure 5 shows boxplots for the significant correlations between prediction accuracy and behaviors (*p* < .05/192) with an effect size of |*r*| > .1. The boxplots are color coded by the category of the behavioral measure and the word clouds at the bottom provide a qualitative representation of the box plots. The complete summary of all the 192 measures can be found in Supplementary Table ST2 and scatterplots of some notable associations are also presented in Supplementary Figure SF8.

We found that task performance, intelligence, emotional attributes, and alertness significantly influenced the ability to discriminate between task and rest based on DFC in three cognitively demanding tasks, that is, working memory, social cognition, and language.

The working memory task identifiability measure was associated with its overall task performance accuracy (*r* = .28, *p* = 2 × 10^−20^) as well as with accuracy of each task condition, such as zero‐back tool (*r* = .28, *p* = 1.5 × 10^−20^); two‐back face (*r* = .21, *p* = 4.9 × 10^−12^); and so forth. Similarly, we also found negative associations with reaction time during the overall task (*r* = −.11, *p* = 2.4 × 10^−4^) and during individual zero‐back conditions like body (*r* = −.13, *p* = 1.4 × 10^−5^); place (*r* = −.17, *p* = 3.3 × 10^−8^); and so forth. From the cognition category, total cognition (*r* = .19, *p* = 5.6 × 10^−10^); fluid cognition (*r* = .17, *p* = 2.7 × 10^−8^); executive functioning (*r* = .17, *p* = 7.6 × 10^−8^); and cognition speed (*r* = .12, *p* = 1.8 × 10^−4^) were some of the measures significantly associated with the task identifiability measure. Motor abilities such as manual dexterity (*r* = .15, *p* = 2.1 × 10^−6^) and physical endurance (*r* = .15, *p* = 5.5 × 10^−7^) were other associated measures. In addition, we found that individuals with traits linked to aggression (*r* = −.14, *p* = 3.8 × 10^−6^) showed weaker modulation of FC dynamics during the task compared to rest.

The social cognition task identifiability measure was significantly associated with its task performance accuracy (*r* = .12, *p* = 2.5 × 10^−4^) and total cognition (*r* = .13, *p* = 3.2 × 10^−5^). It was also associated with accuracy in the working memory task (*r* = .12, *p* = 2.3 × 10^−4^) as well as its two‐back face condition (*r* = .12, *p* = 1.1 × 10^−4^). Furthermore, individuals with lower levels of alertness were found to engage poorly with this task, as indicated by a negative association with sleep quality scores, assessed by the Pittsburgh sleep quality index (PSQI) (*r* = −.14, *p* = 1.1 × 10^−5^). Note that a greater PSQI score indicates poorer quality of sleep.

The language task identifiability measure was significantly correlated with its task performance accuracy (*r* = .2, *p* = 5.5 × 10^−11^) as well as working memory task accuracy (*r* = .18, *p* = 1.4 × 10^−8^). Individuals who perceived greater difficulty in the story (*r* = .17, *p* = 3.5 × 10^−8^) and math (*r* = .17, *p* = 8.8 × 10^−8^) conditions of the task may have tried to engage more, thereby leading to stronger positive associations with the identifiability measure. Significant associations were also found with crystallized cognition (*r* = .12, *p* = 7.8 × 10^−5^); fluid intelligence (*r* = .16, *p* = 1.7 × 10^−7^); language and reading abilities (*r* = .13, *p* = 4.7 × 10^−5^); episodic memory (*r* = .12, *p* = 7.8 × 10^−5^); and physical endurance (*r* = .12, *p* = 1.2 × 10^−4^) among others. One emotion measure, that is, perceived stress (*r* = −.11, *p* = 2.4 × 10^−4^) was negatively correlated with the identifiability measure, indicating that greater susceptibility to stress may diminish task engagement, thereby producing weaker modulation of FC dynamics compared to rest.

To further account for the influence of intelligence and task performance on the associations, we performed partial correlation by regressing out the top principal components of cognition and task performance for each task (see Supplementary Methods SM2), in addition to age and gender. Although the number of surviving measures was reduced, the top associations were still retained. Supplementary Figure SF1 shows the boxplot graphs and corresponding word clouds of statistically significant correlations (with 75% confidence interval) observed after controlling for the influence of age, gender, cognition and task performance, and the Bonferroni correction (*p* < .05/192) with effect size threshold (|*r*| > 0.1).

## 4 DISCUSSION AND CONCLUSION

In this study, we developed a novel methodology known as MTPA and applied it for the first time to 7 different tasks, each performed by more than 1,000 healthy adults, to discriminate task from rest based on DFC specifically calculated from fMRI signals predominantly evoked by internal cognitive states with minimal influence from external stimuli. By employing linear logistic regression, MTPA successfully classified the multivariate temporal patterns of time‐varying functional connectivity from rest and task with greater than 70% accuracy at the brain region level in all the seven tasks, when mean task activation was removed. When these prediction accuracies were summarized into a lower dimension of large‐scale brain networks, they exceeded the threshold in four of the tasks, that is, working memory, social cognition, language, and motor. As a result, these tasks were underpinned by relatively unique functional network architectures. We also demonstrate using MTPA that DFC outperforms static functional connectivity in distinguishing between the cognitive states of task and rest. Furthermore, MTPA enabled the identification of several behavioral measures such as intelligence, perceived stress, aggression, and alertness that were significantly associated with interindividual variation in the discriminability between task and rest based on DFC.

### 4.1 MTPA to discriminate between task and rest

One of the most notable advantages of multivariate classification in fMRI is its increased sensitivity to detect distinct cognitive states from multiple features such as voxels (Haxby et al., 2001; Norman et al., 2006). However, this approach had so far not been applied to decode cognitive states based on temporal patterns of activity. Therefore, when we performed multivariate classification in the temporal domain, it provided us with the sensitivity required to address an important challenge in fMRI research: to unequivocally characterize functional connectivity differences between distinct cognitive states. Moreover, the added sensitivity allowed us to explore the feasibility of distinguishing between the naturally endogenous resting‐state and an intrinsic version of task‐state from which explicit task activation effects are removed. Specifically, MTPA enabled us to establish that distinct cognitive states underlying resting‐state and task engagement can be decoded with greater sensitivity using DFC, compared to time‐averaged connectivity. Importantly, for four of the tasks, we were also able to summarize the results in the spatial domain as task‐specific spatial network configurations formed by above‐threshold intranetwork and internetwork dynamic connections (see Section 3.1, Figure 2—right panel). Furthermore, the probability estimate value of each individual prediction from logistic regression facilitated comparison between DFC and individual behavioral measures.

### 4.2 Network configurations enabling successful discrimination between task and rest based on DFC

Using DFC estimates, MTPA produced several internetwork and intranetwork connections with above‐threshold out‐of‐sample classification accuracies in four tasks. Together, they produced distinct task‐dependent spatial network architectures that successfully discriminated between functional connectivity dynamics of each task and rest, after mean task activation was removed. Distinct cognitive constraints are imposed during task and rest, which lead to differences in the stability of functional connectivity dynamics (Cohen, 2018). Such alterations in stability likely translate to distinct patterns of time‐varying connectivity in the temporal domain. Our current results further suggest that some of these pattern differences stem intrinsically from underlying cognitive states associated with rest and task beyond the brain response produced by the explicitly defined task conditions.

Performing a task involves several interactions in the brain subserving low‐level stimulus processing as well as high‐level complex cognitive processing. While some of these interactions may be time‐locked to task stimuli, others may be occurring intrinsically at different times throughout the task state. Our current analysis likely captures the latter. Connections between visual subnetworks, mainly comprising occipital and fusiform areas, and other high‐level networks in the visual‐based tasks were likely responsible for relaying visual information (Grill‐Spector & Malach, 2004) for the cognitive synthesis of stimuli. In the auditorily presented language task, this function was likely assumed by the auditory areas within the superior and middle temporal lobes (Howard et al., 2000; Xu et al., 2019) covering parts of the DMN subnetwork B. Areas within these temporal lobes have also been implicated in memory, theory of mind, and self‐referential processing (Gallagher & Frith, 2003; Schurz et al., 2014; Xu et al., 2019), thereby explaining their widespread connections in the working memory, social cognition and language tasks. Task performance necessitates executive processing, response selection, and memory, which was likely represented in the connectivity dynamics of the control subnetworks encompassing precuneus and frontotemporal regions (Cavanna & Trimble, 2006; Seeley et al., 2007), in the three cognitively demanding tasks.

Meeting the distinct requirements of each task may have been achieved through dynamic alterations of some specialized connections. The working memory task involved remembering various tools, places, faces, and body parts. This was likely facilitated by the widespread connections of the temporoparietal network with the cognitive subcomponents of the default mode, control, and frontoparietal networks. Objective stimuli recognized in the LOC of the temporoparietal network were likely directed by attentional resources (frontoparietal) toward contextual memory processing subsequently leading to appropriate response selection (control network and DMN). In the social cognition task, processing of animations in the theory‐of‐mind context was likely facilitated by connections associated with the DMN subnetwork B and temporoparietal network (Gallagher & Frith, 2003; Schurz et al., 2014; Xu et al., 2019). Given the role of the salience network in bottom‐up filtering (Menon & Uddin, 2010), their connections with the salience subnetworks may have enabled social inferences via the salient visual features identified from the presented animation. In the language task, the salience subnetwork A, mainly comprising fronto‐insular regions, was the central hub connecting to DMN, somatomotor, control and limbic subnetworks. In this task, participants were required to either perform mental mathematical operations or listen to a story and make high‐level inferences, both of which involve internal contextual processing (DMN) using language and semantics (somatomotor). Furthermore, connections with the limbic subnetwork likely facilitated the identification and auditory cognition of salient emotional components within stories presented during the task (Dixon et al., 2017; Jackson et al., 2018). In the motor task, following cues to move may have evoked the connection between the primary somatomotor and primary visual subnetworks. No other connections exceeded the threshold in this task since it did not necessitate cognitive complexity.

Although the network configurations of language and motor tasks were unique, the extensive involvement of the temporoparietal network in sharing visual information with high‐level cognitive networks explains the overlap of network configurations between the social cognition and working memory tasks, that is, the only successful tasks with visual stimuli.

### 4.3 Comparison between dynamic and static functional connectivity in discriminating between task and rest

To discriminate between task and rest based on region‐to‐region static functional connectivity, we performed one‐dimensional MTPA with logistic regression where the number of timepoint features was equal to one. No network configurations could be recovered for any task and DFC outperformed static functional connectivity in discriminating task from rest (see Section 3.2, Figure 4). Although this difference in performance could also be attributed to larger number of features in MTPA using DFC, our findings are consistent with previous work by Cole et al. (2014) and Fair et al. (2007) where it was demonstrated that static functional network configurations undergo only subtle changes from resting‐state to task‐engagement. Therefore, brain networks most likely accommodate varying task demands by adjusting their functional connectivity dynamics, but this may not necessarily translate to an equivalent alteration in time‐averaged functional connectivity.

However, unlike DFC, the underlying neurophysiological mechanisms (e.g., slow timescale Hebbian synaptic strength processes) potentially supporting time‐averaged connectivity are better known (Mill et al., 2021; Newbold et al., 2020; Petersen & Sporns, 2015). The neurophysiological basis of DFC remains an open issue, despite several recent studies in this area. Recent evidence suggests correlative links between BOLD dynamic FC and transient neuronal synchrony tracking resting as well as task arousal at various time‐scales using different imaging modalities in animal and human studies (Chang et al., 2016; Hutchison, Womelsdorf, Allen, et al., 2013; Hutchison, Womelsdorf, Gati, et al., 2013; Keilholz, 2014; Majeed et al., 2009; Thompson et al., 2013). Notably, transient EEG microstates have been shown to reliably predict dynamic FC of large‐scale brain networks inferred from concurrent 7 T fMRI‐EEG imaging, thus revealing potential electrophysiological biomarkers of distinct recurring FC states (Abreu et al., 2021). Neuropsychiatric and neurological conditions, including schizophrenia and epileptic seizures, have also been characterized by aberrant fMRI connectivity dynamics (Hutchison, Womelsdorf, Allen, et al., 2013; Hutchison, Womelsdorf, Gati, et al., 2013; Kottaram et al., 2018; Liao, Zhang, et al., 2014). Moreover, some studies suggest that entropy changes in neuronal processing complexity (Wang et al., 2018) and neuronal avalanches occurring at discrete time points (Tagliazucchi et al., 2012) may subserve fast‐scale dynamics of functional connectivity. These may be more effectively captured by a TR‐level approach such as FLS. Nonetheless, until there is more definitive empirical evidence explaining the neurophysiological correlates of dynamic connectivity, its apparent superiority in discriminating between task and rest over its comparatively well‐established static counterpart needs to be considered with caution.

### 4.4 Influence of behavior on discriminating between task and rest based on functional connectivity dynamics

Previously, it has been shown that better task performance associates with lower ambiguity in distinguishing between contexts based on DFC (Fong et al., 2019; Gonzalez‐Castillo & Bandettini, 2018; Xie et al., 2019; Zhang et al., 2013). Consistent with these observations, we found that task performance was significantly correlated with the task identifiability measures of three tasks, that is, working memory, social cognition, and language. While interindividual variation in resting‐state DFC can capture the variance in several HCP behavioral measures including intelligence (Liégeois et al., 2019), Schultz and Cole (2016) observed that better task performance and intelligence led to smaller differences between time‐averaged task and rest functional networks due to more efficient network reconfigurations. Contrarily, we found several intelligence measures that were significantly and positively correlated with distinguishability in the three tasks. A likely explanation is that greater intelligence and task performance are characterized by minimal static network reconfigurations because the dynamics of functional connectivity undergo highly efficient temporal pattern modifications with respect to resting state.

Some of the measures were crucial to specific tasks. For instance, attributes like cognition processing speed and fluid intelligence may have been crucial for quick encoding and recall in the working memory task, while crystallized intelligence, episodic memory and language abilities likely determined success in accurately inferring about stories and performing mental math. Additionally, task‐rest discrimination in all the three tasks was associated with the performance in the working memory task, highlighting the task's close relationship with general cognitive capabilities. Specifically, the influence of the two‐back face condition's accuracy on the social cognition task portrays how the ability to remember human faces closely connects with inferring about mental states.

In addition to influencing DFC during task (Tobia et al., 2017), psychosocial stress, negative emotions, and anxiety negatively impact attention (Liston et al., 2009), working memory performance (Luettgau et al., 2018), problem solving (Nair et al., 2020) and executive functioning (Denkova et al., 2010). Therefore, the significant negative correlation between perceived stress and distinguishability between rest and the language task could be explained by the potential interference of stress in the emotional processing components of the story condition. Similarly, individuals prone to aggression may have displayed greater frustration upon increasing cognitive complexity and failure to remember accurately, thereby leading to a significant negative correlation of aggression in the working memory task. In the social cognition task, low levels of alertness based on sleep quality likely interfered with effectively disambiguating social interactions (Gordon et al., 2017), thus explaining the significant negative correlation. We also found that physical endurance was significantly associated with task‐rest distinction in the working memory and language tasks, both of which involved sustained concentration. Physical endurance is closely related to mental toughness (Crust & Clough, 2005), thereby explaining why individuals low on endurance may have “given‐up” earlier in such tasks leading to deteriorating engagement over time, reflected by weaker modulation of FC dynamics.

Therefore, intelligence, task performance, stress, aggression, alertness, and endurance, among others significantly contribute to interindividual variation in the discriminability between task and rest based on functional connectivity dynamics, after the removal of mean task activation. Together, this suggests that greater intelligence and emotional stability lead to stronger engagement with task and hence greater discrimination from rest. This also points toward the potential for using context‐dependent alterations in functional connectivity dynamics to identify individuals with greater susceptibility to neuropsychiatric conditions.

### 4.5 Limitations and future scope

Although MTPA provides a promising avenue for distinguishing between task and rest DFC, this work has some limitations. Summarizing the connections reduced the overall prediction accuracy and resolution of interpretation at the cost of robustness. Despite no above‐threshold network‐level connections in the gambling, emotion, and relational tasks, we found successful region‐level connections. For instance, in the relational task, connections between the visual processing occipital and high‐level frontal regions may have facilitated the visual cognition necessary to match intricate patterns. On the contrary, there were very few surviving connections in the emotion and gambling tasks. Greater task complexity producing larger interindividual variability in DFC could be a possible cause. Di and Biswal (2019) similarly failed to find any statistically significant group‐level connections that were dynamically modulated by the condition blocks of these same HCP tasks through psychophysiological interactions (PPI). Additionally, our initial 150‐region parcellation did not include all subcortical structures since we mainly focused on cortical dynamics. As a result, we may have missed some crucial connections pertaining to tasks like emotion processing where the amygdala plays a key role (Barch et al., 2013). The overall performance of MTPA could improve further with higher fMRI signal to noise ratio, nonlinear pattern separation and differences in classifier, parcellation scheme or method of DFC estimation. Therefore, we recommend that future studies explore MTPA with different parameters and tasks to assess its reliability. Although we find that dynamic FC outperforms static FC with MTPA, the substantial difference in the feature dimensionalities between the two could be a major factor driving the difference in classification performance. Moreover, a cautious interpretation is essential given that the neurophysiological underpinnings of dynamic FC remain unclear. Future work involving invasive animal studies with naturalistic stimuli could further elucidate the neurophysiological correlates of dynamic FC. In general, time‐resolved connectivity readily lends itself to analyses like the MTPA, compared to its more rigid time‐averaged counterpart. The TR‐level approach is one of many available methods used to estimate dynamic FC in fMRI (Preti et al., 2017). Although we have used this approach in our current MTPA work, there is scope for future work to explore MTPA with other established dynamic FC methods, including sliding‐window analyses, where successive window FC estimates are used as classification features. This also highlights the flexibility of our proposed MTPA method. Furthermore, the reliability of MTPA depends on sample size. While it is suitable for large datasets, the amount of training data might be insufficient to produce reasonable out‐of‐sample prediction with smaller sample sizes that are typical of fMRI acquisitions.

Distinguishing between the temporal patterns of dynamic FC associated with two different tasks (task A vs. task B) would be an interesting future avenue of investigation and may enable characterization of similarities and distinctions between tasks. Similarly, connections enabling discrimination between DFC patterns associated with healthy and pathological populations could offer further insights into biomarkers and treatments. Instead of regressing task activation based on the conventional HRF‐convolved GLM, finite impulse response modeling should be considered in future studies, given that it may be able to better model task activations (Cole et al., 2019). Although we have briefly explored the classification weights from MTPA to analyze their relationship with task conditions (see Supplementary Results SR2), there is still scope to investigate further in this regard. Similarly, investigating the specific behaviors underlying the classification in individual region‐to‐region or network‐to‐network connections instead of using an average task identifiability measure is another interesting direction worth exploring. Finally, we have also explored the utility of MTPA as a predictive tool with minimally preprocessed data uncorrected for confounds and task activation effects (see Supplementary Analysis SA1). Following this predictive approach, MTPA can be flexibly adopted in the future to investigate the relative sensitivity of functional connections to neural signals and nonneural confounds in terms of their dynamics in specific contexts. Similarly, individual‐specific behaviors most susceptible to neural and nonneural dynamics can be delineated, and cautiously considered while recruiting for future task paradigms. The predictive utility of MTPA could also lead to the development of diagnostic capabilities in neuropsychiatry research.

### 4.6 Conclusion

Changing cognitive contexts associated with resting‐state and task engagement are characterized better by dynamic adaptations of functional brain networks than static characterizations. Through our proposed method of MTPA, these dynamic adaptations can be reliably captured as linearly separable temporal patterns from large sample sizes in multiple tasks. Notably, tasks modulate functional connectivity dynamics to varying extents across individuals and an individual's intelligence, alertness, endurance, and emotional stability can influence the extent of this modulation. This highlights the importance of modeling interindividual variation in behavioral measures when investigating context‐dependent DFC in future task fMRI studies.

## CONFLICT OF INTEREST

The authors declare no potential conflicts of interest.

## Supporting information


**Appendix S1:** Supplementary Information

## Data Availability

All data used in the present study are available for download from the Human Connectome Project (www.humanconnectome.org). Users must agree to data use terms for the HCP before being allowed access to the data and ConnectomeDB, details are provided at https://www.humanconnectome.org/study/hcp-young-adult/data-use-terms. The HCP has implemented a two‐tiered plan for data sharing, with different provisions for handling open access data and restricted data (e.g., data related to family structure, age by year, etc.). Both open access and restricted data were utilized in the present study. All results from the present study are available upon request to the author.

## References

[hbm25732-bib-0001] Abreu, R. , Jorge, J. , Leal, A. , Koenig, T. , & Figueiredo, P. (2021). EEG microstates predict concurrent fMRI dynamic functional connectivity states. Brain Topography, 34(1), 41–55. 10.1007/s10548-020-00805-1 33161518

[hbm25732-bib-0002] Allen, E. A. , Damaraju, E. , Plis, S. M. , Erhardt, E. B. , Eichele, T. , & Calhoun, V. D. (2014). Tracking whole‐brain connectivity dynamics in the resting state. Cerebral Cortex, 24(3), 663–676. 10.1093/cercor/bhs352 23146964 PMC3920766

[hbm25732-bib-0003] Barch, D. M. , Burgess, G. C. , Harms, M. P. , Petersen, S. E. , Schlaggar, B. L. , Corbetta, M. , … WU‐Minn Human Connectome . (2013). Function in the human connectome: Task‐fMRI and individual differences in behavior. NeuroImage, 80, 169–189. 10.1016/j.neuroimage.2013.05.033 23684877 PMC4011498

[hbm25732-bib-0004] Biswal, B. B. , Van Kylen, J. , & Hyde, J. S. (1997). Simultaneous assessment of flow and BOLD signals in resting‐state functional connectivity maps. NMR in Biomedicine, 10(4–5), 165–170. 10.1002/(sici)1099-1492(199706/08)10:4/5<165::aid-nbm454>3.0.co;2-7 9430343

[hbm25732-bib-0005] Bolt, T. , Nomi, J. S. , Rubinov, M. , & Uddin, L. Q. (2017). Correspondence between evoked and intrinsic functional brain network configurations. Human Brain Mapping, 38(4), 1992–2007. 10.1002/hbm.23500 28052450 PMC6866760

[hbm25732-bib-0006] Bolton, T. A. W. & Ville, D. V. D. (2020). Dynamics of brain activity captured by graph signal processing of neuroimaging data to predict human behaviour. Paper presented at the 2020 IEEE 17th International Symposium on Biomedical Imaging (ISBI).

[hbm25732-bib-0007] Calhoun, V. D. , Kiehl, K. A. , & Pearlson, G. D. (2008). Modulation of temporally coherent brain networks estimated using ICA at rest and during cognitive tasks. Human Brain Mapping, 29(7), 828–838. 10.1002/hbm.20581 18438867 PMC2649823

[hbm25732-bib-0008] Cavanna, A. E. , & Trimble, M. R. (2006). The precuneus: A review of its functional anatomy and behavioural correlates. Brain, 129(3), 564–583. 10.1093/brain/awl004 16399806

[hbm25732-bib-0009] Chang, C. , Leopold, D. A. , Schölvinck, M. L. , Mandelkow, H. , Picchioni, D. , Liu, X. , … Duyn, J. H. (2016). Tracking brain arousal fluctuations with fMRI. Proceedings of the National Academy of Sciences of the United States of America, 113(16), 4518–4523. 10.1073/pnas.1520613113 27051064 PMC4843437

[hbm25732-bib-0010] Cheng, L. , Zhu, Y. , Sun, J. , Deng, L. , He, N. , Yang, Y. , … Tong, S. (2018). Principal states of dynamic functional connectivity reveal the link between resting‐state and task‐state brain: An fMRI study. International Journal of Neural Systems, 28(7), 1850002. 10.1142/s0129065718500028 29607681

[hbm25732-bib-0011] Cohen, J. R. (2018). The behavioral and cognitive relevance of time‐varying, dynamic changes in functional connectivity. NeuroImage, 180(Pt B), 515–525. 10.1016/j.neuroimage.2017.09.036 28942061 PMC6056319

[hbm25732-bib-0012] Cole, M. W. , Bassett, D. S. , Power, J. D. , Braver, T. S. , & Petersen, S. E. (2014). Intrinsic and task‐evoked network architectures of the human brain. Neuron, 83(1), 238–251. 10.1016/j.neuron.2014.05.014 24991964 PMC4082806

[hbm25732-bib-0013] Cole, M. W. , Ito, T. , Schultz, D. , Mill, R. , Chen, R. , & Cocuzza, C. (2019). Task activations produce spurious but systematic inflation of task functional connectivity estimates. NeuroImage, 189, 1–18. 10.1016/j.neuroimage.2018.12.054 30597260 PMC6422749

[hbm25732-bib-0014] Craddock, R. C. , James, G. A. , Holtzheimer, P. E., 3rd , Hu, X. P. , & Mayberg, H. S. (2012). A whole brain fMRI atlas generated via spatially constrained spectral clustering. Human Brain Mapping, 33(8), 1914–1928. 10.1002/hbm.21333 21769991 PMC3838923

[hbm25732-bib-0015] Crust, L. , & Clough, P. J. (2005). Relationship between mental toughness and physical endurance. Perceptual and Motor Skills, 100(1), 192–194. 10.2466/pms.100.1.192-194 15773710

[hbm25732-bib-0016] Denkova, E. , Nomi, J. S. , Uddin, L. Q. , & Jha, A. P. (2019). Dynamic brain network configurations during rest and an attention task with frequent occurrence of mind wandering. Human Brain Mapping, 40(15), 4564–4576. 10.1002/hbm.24721 31379120 PMC6865814

[hbm25732-bib-0017] Denkova, E. , Wong, G. , Dolcos, S. , Sung, K. , Wang, L. , Coupland, N. , & Dolcos, F. (2010). The impact of anxiety‐inducing distraction on cognitive performance: A combined brain imaging and personality investigation. PLoS One, 5(11), e14150. 10.1371/journal.pone.0014150 21152391 PMC2994755

[hbm25732-bib-0018] DeSalvo, M. N. , Douw, L. , Takaya, S. , Liu, H. , & Stufflebeam, S. M. (2014). Task‐dependent reorganization of functional connectivity networks during visual semantic decision making. Brain and Behavior, 4(6), 877–885. 10.1002/brb3.286 25365802 PMC4178300

[hbm25732-bib-0019] Di, X. , & Biswal, B. B. (2019). Toward task connectomics: Examining whole‐brain task modulated connectivity in different task domains. Cerebral Cortex, 29(4), 1572–1583. 10.1093/cercor/bhy055 29931116 PMC7302740

[hbm25732-bib-0020] Dixon, M. L. , Thiruchselvam, R. , Todd, R. , & Christoff, K. (2017). Emotion and the prefrontal cortex: An integrative review. Psychological Bulletin, 143(10), 1033–1081. 10.1037/bul0000096 28616997

[hbm25732-bib-0021] Elton, A. , & Gao, W. (2015). Task‐related modulation of functional connectivity variability and its behavioral correlations. Human Brain Mapping, 36(8), 3260–3272. 10.1002/hbm.22847 26015070 PMC6869497

[hbm25732-bib-0022] Fair, D. A. , Schlaggar, B. L. , Cohen, A. L. , Miezin, F. M. , Dosenbach, N. U. , Wenger, K. K. , … Petersen, S. E. (2007). A method for using blocked and event‐related fMRI data to study "resting state" functional connectivity. NeuroImage, 35(1), 396–405. 10.1016/j.neuroimage.2006.11.051 17239622 PMC2563954

[hbm25732-bib-0023] Fan, R.‐E. , Chang, K.‐W. , Hsieh, C.‐J. , Wang, X.‐R. , & Lin, C.‐J. (2008). LIBLINEAR: A library for large linear classification. Journal of Machine Learning Research, 9(Aug), 1871–1874.

[hbm25732-bib-0024] Finn, E. S. , Glerean, E. , Khojandi, A. Y. , Nielson, D. , Molfese, P. J. , Handwerker, D. A. , & Bandettini, P. A. (2020). Idiosynchrony: From shared responses to individual differences during naturalistic neuroimaging. NeuroImage, 215, 116828. 10.1016/j.neuroimage.2020.116828 32276065 PMC7298885

[hbm25732-bib-0025] Fong, A. H. C. , Yoo, K. , Rosenberg, M. D. , Zhang, S. , Li, C. R. , Scheinost, D. , … Chun, M. M. (2019). Dynamic functional connectivity during task performance and rest predicts individual differences in attention across studies. NeuroImage, 188, 14–25. 10.1016/j.neuroimage.2018.11.057 30521950 PMC6401236

[hbm25732-bib-0026] Fox, M. D. , & Raichle, M. E. (2007). Spontaneous fluctuations in brain activity observed with functional magnetic resonance imaging. Nature Reviews Neuroscience, 8(9), 700–711. 10.1038/nrn2201 17704812

[hbm25732-bib-0027] Gallagher, H. L. , & Frith, C. D. (2003). Functional imaging of ‘theory of mind’. Trends in Cognitive Sciences, 7(2), 77–83. 10.1016/S1364-6613(02)00025-6 12584026

[hbm25732-bib-0028] Glasser, M. F. , Sotiropoulos, S. N. , Wilson, J. A. , Coalson, T. S. , Fischl, B. , Andersson, J. L. , … Jenkinson, M. (2013). The minimal preprocessing pipelines for the Human Connectome Project. NeuroImage, 80, 105–124. 10.1016/j.neuroimage.2013.04.127 23668970 PMC3720813

[hbm25732-bib-0029] Gonzalez‐Castillo, J. , & Bandettini, P. A. (2018). Task‐based dynamic functional connectivity: Recent findings and open questions. NeuroImage, 180(Pt B), 526–533. 10.1016/j.neuroimage.2017.08.006 28780401 PMC5797523

[hbm25732-bib-0030] Gordon, A. M. , Mendes, W. B. , & Prather, A. A. (2017). The social side of sleep: Elucidating the links between sleep and social processes. Current Directions in Psychological Science, 26(5), 470–475. 10.1177/0963721417712269 29398789 PMC5791747

[hbm25732-bib-0031] Grill‐Spector, K. , & Malach, R. (2004). The human visual cortex. Annual Review of Neuroscience, 27, 649–677. 10.1146/annurev.neuro.27.070203.144220 15217346

[hbm25732-bib-0032] Haxby, J. V. , Gobbini, M. I. , Furey, M. L. , Ishai, A. , Schouten, J. L. , & Pietrini, P. (2001). Distributed and overlapping representations of faces and objects in ventral temporal cortex. Science, 293(5539), 2425–2430. 10.1126/science.1063736 11577229

[hbm25732-bib-0033] Howard, M. A. , Volkov, I. O. , Mirsky, R. , Garell, P. C. , Noh, M. D. , Granner, M. , … Brugge, J. F. (2000). Auditory cortex on the human posterior superior temporal gyrus. Journal of Comparative Neurology, 416(1), 79–92. 10.1002/(sici)1096-9861(20000103)416:1<79::Aid-cne6>3.0.Co;2-2 10578103

[hbm25732-bib-0034] Hutchison, R. M. , Womelsdorf, T. , Allen, E. A. , Bandettini, P. A. , Calhoun, V. D. , Corbetta, M. , … Chang, C. (2013). Dynamic functional connectivity: Promise, issues, and interpretations. NeuroImage, 80, 360–378. 10.1016/j.neuroimage.2013.05.079 23707587 PMC3807588

[hbm25732-bib-0035] Hutchison, R. M. , Womelsdorf, T. , Gati, J. S. , Everling, S. , & Menon, R. S. (2013). Resting‐state networks show dynamic functional connectivity in awake humans and anesthetized macaques. Human Brain Mapping, 34(9), 2154–2177. 10.1002/hbm.22058 22438275 PMC6870538

[hbm25732-bib-0036] Iraji, A. , Miller, R. , Adali, T. , & Calhoun, V. D. (2020). Space: A missing piece of the dynamic puzzle. Trends in Cognitive Sciences, 24(2), 135–149. 10.1016/j.tics.2019.12.004 31983607 PMC7809367

[hbm25732-bib-0037] Jackson, R. L. , Bajada, C. J. , Rice, G. E. , Cloutman, L. L. , & Lambon Ralph, M. A. (2018). An emergent functional parcellation of the temporal cortex. NeuroImage, 170, 385–399. 10.1016/j.neuroimage.2017.04.024 28419851

[hbm25732-bib-0038] Kalaba, R. , & Tesfatsion, L. (1989). Time‐varying linear regression via flexible least squares. Computers & Mathematics with Applications, 17(8), 1215–1245. 10.1016/0898-1221(89)90091-6

[hbm25732-bib-0040] Keilholz, S. D. (2014). The neural basis of time‐varying resting‐state functional connectivity. Brain Connectivity, 4(10), 769–779. 10.1089/brain.2014.0250 24975024 PMC4268576

[hbm25732-bib-0041] Kottaram, A. , Johnston, L. , Ganella, E. , Pantelis, C. , Kotagiri, R. , & Zalesky, A. (2018). Spatio‐temporal dynamics of resting‐state brain networks improve single‐subject prediction of schizophrenia diagnosis. Human Brain Mapping, 39(9), 3663–3681. 10.1002/hbm.24202 29749660 PMC6866493

[hbm25732-bib-0042] Kucyi, A. , & Davis, K. D. (2014). Dynamic functional connectivity of the default mode network tracks daydreaming. NeuroImage, 100, 471–480. 10.1016/j.neuroimage.2014.06.044 24973603

[hbm25732-bib-0043] Laumann, T. O. , Snyder, A. Z. , Mitra, A. , Gordon, E. M. , Gratton, C. , Adeyemo, B. , … Petersen, S. E. (2017). On the stability of BOLD fMRI correlations. Cerebral Cortex, 27(10), 4719–4732. 10.1093/cercor/bhw265 27591147 PMC6248456

[hbm25732-bib-0044] Liao, W. , Wu, G. R. , Xu, Q. , Ji, G. J. , Zhang, Z. , Zang, Y. F. , & Lu, G. (2014). DynamicBC: A MATLAB toolbox for dynamic brain connectome analysis. Brain Connectivity, 4(10), 780–790. 10.1089/brain.2014.0253 25083734 PMC4268585

[hbm25732-bib-0045] Liao, W. , Zhang, Z. , Mantini, D. , Xu, Q. , Ji, G. J. , Zhang, H. , … Lu, G. (2014). Dynamical intrinsic functional architecture of the brain during absence seizures. Brain Structure & Function, 219(6), 2001–2015. 10.1007/s00429-013-0619-2 23913255

[hbm25732-bib-0046] Liégeois, R. , Li, J. , Kong, R. , Orban, C. , Van De Ville, D. , Ge, T. , … Yeo, B. T. T. (2019). Resting brain dynamics at different timescales capture distinct aspects of human behavior. Nature Communications, 10(1), 2317. 10.1038/s41467-019-10317-7 PMC6534566 31127095

[hbm25732-bib-0047] Linden, D. E. J. , Prvulovic, D. , Formisano, E. , Völlinger, M. , Zanella, F. E. , Goebel, R. , & Dierks, T. (1999). The functional neuroanatomy of target detection: An fMRI study of visual and auditory oddball tasks. Cerebral Cortex, 9(8), 815–823. 10.1093/cercor/9.8.815 10601000

[hbm25732-bib-0048] Liston, C. , McEwen, B. S. , & Casey, B. J. (2009). Psychosocial stress reversibly disrupts prefrontal processing and attentional control. Proceedings of the National Academy of Sciences of the United States of America, 106(3), 912–917. 10.1073/pnas.0807041106 19139412 PMC2621252

[hbm25732-bib-0049] Luettgau, L. , Schlagenhauf, F. , & Sjoerds, Z. (2018). Acute and past subjective stress influence working memory and related neural substrates. Psychoneuroendocrinology, 96, 25–34. 10.1016/j.psyneuen.2018.05.036 29879562

[hbm25732-bib-0050] Majeed, W. , Magnuson, M. , & Keilholz, S. D. (2009). Spatiotemporal dynamics of low frequency fluctuations in BOLD fMRI of the rat. Journal of Magnetic Resonance Imaging, 30(2), 384–393. 10.1002/jmri.21848 19629982 PMC2758521

[hbm25732-bib-0051] Meer, J. N. V. , Breakspear, M. , Chang, L. J. , Sonkusare, S. , & Cocchi, L. (2020). Movie viewing elicits rich and reliable brain state dynamics. Nature Communications, 11(1), 5004. 10.1038/s41467-020-18717-w PMC7536385 33020473

[hbm25732-bib-0052] Menon, V. , & Uddin, L. Q. (2010). Saliency, switching, attention and control: A network model of insula function. Brain Structure & Function, 214(5–6), 655–667. 10.1007/s00429-010-0262-0 20512370 PMC2899886

[hbm25732-bib-0053] Mill, R. D. , Hamilton, J. L. , Winfield, E. C. , Lalta, N. , Chen, R. H. & Cole, M. W. (2021). Emergence of task information from dynamic network interactions in the human brain. bioRxiv, 2021.2001.2026.428276. doi:10.1101/2021.01.26.428276

[hbm25732-bib-0054] Mill, R. D. , Ito, T. , & Cole, M. W. (2017). From connectome to cognition: The search for mechanism in human functional brain networks. NeuroImage, 160, 124–139. 10.1016/j.neuroimage.2017.01.060 28131891 PMC5529276

[hbm25732-bib-0055] Nair, N. , Hegarty, J. P., 2nd , Ferguson, B. J. , Hecht, P. M. , Tilley, M. , Christ, S. E. , & Beversdorf, D. Q. (2020). Effects of stress on functional connectivity during problem solving. NeuroImage, 208, 116407. 10.1016/j.neuroimage.2019.116407 31809888

[hbm25732-bib-0056] Newbold, D. J. , Laumann, T. O. , Hoyt, C. R. , Hampton, J. M. , Montez, D. F. , Raut, R. V. , … Dosenbach, N. U. F. (2020). Plasticity and spontaneous activity pulses in disused human brain circuits. Neuron, 107(3), 580–589.e586. 10.1016/j.neuron.2020.05.007 32778224 PMC7419711

[hbm25732-bib-0057] Norman, K. A. , Polyn, S. M. , Detre, G. J. , & Haxby, J. V. (2006). Beyond mind‐reading: Multi‐voxel pattern analysis of fMRI data. Trends in Cognitive Sciences, 10(9), 424–430. 10.1016/j.tics.2006.07.005 16899397

[hbm25732-bib-0058] Ou, J. , Xie, L. , Jin, C. , Li, X. , Zhu, D. , Jiang, R. , … Liu, T. (2015). Characterizing and differentiating brain state dynamics via hidden Markov models. Brain Topography, 28(5), 666–679. 10.1007/s10548-014-0406-2 25331991 PMC4405424

[hbm25732-bib-0059] Petersen, S. E. , & Sporns, O. (2015). Brain networks and cognitive architectures. Neuron, 88(1), 207–219. 10.1016/j.neuron.2015.09.027 26447582 PMC4598639

[hbm25732-bib-0060] Preti, M. G. , Bolton, T. A. , & Van De Ville, D. (2017). The dynamic functional connectome: State‐of‐the‐art and perspectives. NeuroImage, 160, 41–54. 10.1016/j.neuroimage.2016.12.061 28034766

[hbm25732-bib-0061] Schultz, D. H. , & Cole, M. W. (2016). Higher intelligence is associated with less task‐related brain network reconfiguration. The Journal of Neuroscience, 36(33), 8551–8561. 10.1523/jneurosci.0358-16.2016 27535904 PMC4987432

[hbm25732-bib-0062] Schurz, M. , Radua, J. , Aichhorn, M. , Richlan, F. , & Perner, J. (2014). Fractionating theory of mind: A meta‐analysis of functional brain imaging studies. Neuroscience & Biobehavioral Reviews, 42, 9–34. 10.1016/j.neubiorev.2014.01.009 24486722

[hbm25732-bib-0063] Seeley, W. W. , Menon, V. , Schatzberg, A. F. , Keller, J. , Glover, G. H. , Kenna, H. , … Greicius, M. D. (2007). Dissociable intrinsic connectivity networks for salience processing and executive control. The Journal of Neuroscience, 27(9), 2349–2356. 10.1523/jneurosci.5587-06.2007 17329432 PMC2680293

[hbm25732-bib-0064] Shi, L. , Sun, J. , Wu, X. , Wei, D. , Chen, Q. , Yang, W. , … Qiu, J. (2018). Brain networks of happiness: Dynamic functional connectivity among the default, cognitive and salience networks relates to subjective well‐being. Social Cognitive and Affective Neuroscience, 13(8), 851–862. 10.1093/scan/nsy059 30016499 PMC6123521

[hbm25732-bib-0065] Smith, S. M. , Beckmann, C. F. , Andersson, J. , Auerbach, E. J. , Bijsterbosch, J. , Douaud, G. , … WU‐Minn Human Connectome . (2013). Resting‐state fMRI in the Human Connectome Project. NeuroImage, 80, 144–168. 10.1016/j.neuroimage.2013.05.039 23702415 PMC3720828

[hbm25732-bib-0066] Tagliazucchi, E. , Balenzuela, P. , Fraiman, D. , & Chialvo, D. (2012). Criticality in large‐scale brain fMRI dynamics unveiled by a novel point process analysis. Frontiers in Physiology, 3(15), 1–12. 10.3389/fphys.2012.00015 22347863 PMC3274757

[hbm25732-bib-0067] Thompson, G. J. , Merritt, M. D. , Pan, W.‐J. , Magnuson, M. E. , Grooms, J. K. , Jaeger, D. , & Keilholz, S. D. (2013). Neural correlates of time‐varying functional connectivity in the rat. NeuroImage, 83, 826–836. 10.1016/j.neuroimage.2013.07.036 23876248 PMC3815981

[hbm25732-bib-0068] Tobia, M. J. , Hayashi, K. , Ballard, G. , Gotlib, I. H. , & Waugh, C. E. (2017). Dynamic functional connectivity and individual differences in emotions during social stress. Human Brain Mapping, 38(12), 6185–6205. 10.1002/hbm.23821 28940859 PMC6866845

[hbm25732-bib-0069] Tomasi, D. , Wang, R. , Wang, G. J. , & Volkow, N. D. (2014). Functional connectivity and brain activation: A synergistic approach. Cerebral Cortex, 24(10), 2619–2629. 10.1093/cercor/bht119 23645721 PMC4229895

[hbm25732-bib-0070] van den Heuvel, M. P. , & Hulshoff Pol, H. E. (2010). Exploring the brain network: A review on resting‐state fMRI functional connectivity. European Neuropsychopharmacology, 20(8), 519–534. 10.1016/j.euroneuro.2010.03.008 20471808

[hbm25732-bib-0071] Viviano, R. P. , Raz, N. , Yuan, P. , & Damoiseaux, J. S. (2017). Associations between dynamic functional connectivity and age, metabolic risk, and cognitive performance. Neurobiology of Aging, 59, 135–143. 10.1016/j.neurobiolaging.2017.08.003 28882422 PMC5679403

[hbm25732-bib-0072] Wang, D. J. J. , Jann, K. , Fan, C. , Qiao, Y. , Zang, Y. , Lu, H. , & Yang, Y. (2018). Neurophysiological basis of multi‐scale entropy of brain complexity and its relationship with functional connectivity. Frontiers in Neuroscience, 12(352), 1–14. 10.3389/fnins.2018.00352 29896081 PMC5986880

[hbm25732-bib-0073] Wu, X. , He, H. , Shi, L. , Xia, Y. , Zuang, K. , Feng, Q. , … Qiu, J. (2019). Personality traits are related with dynamic functional connectivity in major depression disorder: A resting‐state analysis. Journal of Affective Disorders, 245, 1032–1042. 10.1016/j.jad.2018.11.002 30699845

[hbm25732-bib-0074] Xie, H. , Gonzalez‐Castillo, J. , Handwerker, D. A. , Bandettini, P. A. , Calhoun, V. D. , Chen, G. , … Mitra, S. (2019). Time‐varying whole‐brain functional network connectivity coupled to task engagement. Network Neuroscience, 3(1), 49–66. 10.1162/netn_a_00051 30793073 PMC6326730

[hbm25732-bib-0075] Xu, J. , Lyu, H. , Li, T. , Xu, Z. , Fu, X. , Jia, F. , … Hu, Q. (2019). Delineating functional segregations of the human middle temporal gyrus with resting‐state functional connectivity and coactivation patterns. Human Brain Mapping, 40(18), 5159–5171. 10.1002/hbm.24763 31423713 PMC6865466

[hbm25732-bib-0076] Yeo, B. T. T. , Krienen, F. M. , Sepulcre, J. , Sabuncu, M. R. , Lashkari, D. , Hollinshead, M. , … Buckner, R. L. (2011). The organization of the human cerebral cortex estimated by intrinsic functional connectivity. Journal of Neurophysiology, 106(3), 1125–1165. 10.1152/jn.00338.2011 21653723 PMC3174820

[hbm25732-bib-0077] Zalesky, A. , Fornito, A. , Cocchi, L. , Gollo, L. L. , & Breakspear, M. (2014). Time‐resolved resting‐state brain networks. Proceedings of the National Academy of Sciences of the United States of America, 111(28), 10341–10346. 10.1073/pnas.1400181111 24982140 PMC4104861

[hbm25732-bib-0078] Zhang, X. , Guo, L. , Li, X. , Zhang, T. , Zhu, D. , Li, K. , … Liu, T. (2013). Characterization of task‐free and task‐performance brain states via functional connectome patterns. Medical Image Analysis, 17(8), 1106–1122. 10.1016/j.media.2013.07.003 23938590 PMC3956081

[hbm25732-bib-0079] Zhuang, X. , Yang, Z. , Mishra, V. , Sreenivasan, K. , Bernick, C. , & Cordes, D. (2020). Single‐scale time‐dependent window‐sizes in sliding‐window dynamic functional connectivity analysis: A validation study. NeuroImage, 220, 117111. 10.1016/j.neuroimage.2020.117111 32615255 PMC7594665

