# Supplementary material for: Multi‐timepoint pattern analysis: Influence of personality and behavior on decoding context‐dependent brain connectivity dynamics
Source: Hum Brain Mapp. 2021 Dec 3;43(4):1403–18. doi: 10.1002/hbm.25732 (PMC8837593; doi:10.1002/hbm.25732)
Supplement: Supplementary file 1 — Appendix S1: Supplementary Information [file HBM-43-1403-s001.docx]

Supplementary Material

Multi-Timepoint Pattern Analysis: Influence of personality and behavior on decoding context-dependent brain connectivity dynamics

Table of Contents

[Table ST1 2](#_Toc82522253)

[Supplemental Methods SM1: Linear logistic regression 3](#_Toc82522254)

[Supplemental Methods SM2: Partial correlation controlling for age, sex, cognition & task performance. 4](#_Toc82522255)

[*Figure SF1* 6](#_Toc82522256)

[Supplemental results SR1: Brain visualization of top region-level accuracies 6](#_Toc82522257)

[*Figure SF2* 8](#_Toc82522258)

[*Figure SF3* 8](#_Toc82522259)

[Supplemental results SR2: Relationship between classification weights and task experimental design 9](#_Toc82522260)

[*Figure SF4* 10](#_Toc82522261)

[Figure SF5 11](#_Toc82522262)

[Figure SF6 12](#_Toc82522263)

[Figure SF7 14](#_Toc82522264)

[Figure SF8 16](#_Toc82522265)

[Supplemental Analysis SA1: MTPA using minimally pre-processed data - prior to removal of head motion, physiological and task activation effects 17](#_Toc82522266)

[*Figure SF10* 20](#_Toc82522267)

[Network configurations enabling successful discrimination between task and rest based on dynamic functional connectivity 20](#_Toc82522268)

[Classifying task and rest using static functional connectivity 22](#_Toc82522269)

[*Figure SF11* 23](#_Toc82522270)

[Impact of behavior on classifying task and rest using functional connectivity dynamics 24](#_Toc82522271)

[*Figure SF12* 26](#_Toc82522272)

[*Figure SF13* 28](#_Toc82522273)

[Conclusion 29](#_Toc82522274)

[Table ST2 31](#_Toc82522275)

[Supplemental References 42](#_Toc82522276)

*Table ST1: A summary of the experimental designs and the subjects included from the 7 HCP tasks considered in this study. Note: 1 TR = 720 ms.*

| **Task** | **No. of Subjects (n)** | **Gender**  **(M/F)** | **Scan Length (TRs)** | **Stimuli Modality** | **Subject Response Modality** | **Task Summary** |
| --- | --- | --- | --- | --- | --- | --- |
| Social Cognition | 1000 | 472/549 | 274 | Visual | Verbal | Decide if interaction between moving shapes is SOCIAL or RANDOM |
| Working Memory | 1036 | 492/574 | 405 | Visual | Verbal | Recognize tools, places, faces and body parts from memory (0-back and 2-back). |
| Language | 1017 | 479/541 | 316 | Auditory | Button Push | Listen to STORY and make inference; or perform MATH calculations. |
| Relational Processing | 1000 | 469/545 | 232 | Visual | Verbal | Decide if two presented objects are RELATED in texture or shape; or MATCH objects with the same texture or shape. |
| Motor | 1052 | 491/574 | 284 | Visual | Perform Activity | Follow the cue and MOVE a hand, foot or tongue |
| Emotion Processing^†^ | 1000 | 470/546 | 176 | Visual | Verbal | Match FACES with expressions (angry or fearful); or match SHAPES |
| Gambling | 1037 | 480/583 | 253 | Visual | Button Push | Guess the range of numbers on a card to win or lose in a REWARD, LOSS or neutral trial. |

† *The last 3 trials of the last task block of each run have not been included in the emotion processing task, due to a bug in the E-prime script. Therefore, the scan length was cut short to 176 TRs (Barch et al., 2013).*

# Supplemental Methods SM1: Linear logistic regression

For MTPA, we implemented L2-regularized linear logistic regression using the LIBLINEAR tool (Fan et al., 2008), which solves the following equation:

$\min_{w} {[\frac{1}{2}\mathbf{w}}^{T}\mathbf{w}+C\sum_{i=1}^{n} log(1+e^{-b\left( i \right)w^{T}a\left( i \right)})]$- (1s)

Given a time course of DFC β coefficients with associated labels of task or rest (a(i), b(i)), i = 1 . . . n, with n being total number of samples in the training dataset, a(i) ∈ R_n_, b(i) ∈ {0,1}, **w** is the weight parameter vector, C > 0 is the cost term, and $\log\left( 1+e^{-b\left( i \right)w^{T}a\left( i \right)} \right)$ is the loss function derived from a probabilistic model (Fan et al., 2008). The 10-fold cross-validation procedure involves randomly splitting the training dataset into 10 folds iteratively and developing classification models by training on 9 folds and testing on one. To obtain each cross-validation model, the algorithm solves eq. *(1s)* by optimising the regularisation cost term C. At the end of the cross-validation procedure, the C associated with most optimal predictive model is selected and subsequently used to train and test a model on the full dataset. The classification model also outputs a probability estimate for each prediction. The probability estimate of the predicted label for the vector of features *a* is given by the sigmoid function $\left[ \frac{1}{1+e^{-w^{T}a}} \right]$, where *w* is the feature weight vector. This represents the probability of an instance belonging to one label or the other. For probability estimates ≥ 0.5, the assigned label is 1 (task) and otherwise 0 (rest).

# Supplemental Methods SM2: Partial correlation controlling for age, sex, cognition & task performance.

To eliminate confounding effects of cognitive performance and in-scanner task performance on the associations of the 192 HCP measures with the three HCP tasks, i.e., working memory, social cognition, and language, we performed an additional analysis by partialling out the effect of cognition and in-scanner task performance, in addition to age and sex. Summary measures of cognition and task performance were derived using Principal Component Analysis (PCA). Separate PCAs was performed to reduce the dimensionality of 38 cognition measures, 54 working memory task measures, 21 social cognition task measures, and 8 language task measures. The top cognition PC accounted for 99.76% of the explained variance. On the other hand, the top two working memory PCs accounted for 47.9% and 10.4%, top two social cognition PCs for 56% and 27.3%, and top two language PCs for 75.1% and 24.8% of the explained variances. Therefore, cognition was represented by its top PC alone, whereas the rest of the measures were represented by their top two PCs to ensure that a reasonable amount of associated variance was partialled out. The results of this analysis have been shown in Supplementary Figure SF1.

**

*Figure SF1: Qualitative and quantitative representations of the significant associations between behavioral measures and task identifiability measures after controlling for the influence of age, gender, intelligence, and task performance. 1) Boxplots showing statistically significant correlations (p < 0.05, |r| > 0.1, Bonferroni corrected), with 75% confidence interval, between individual task identifiability measures and behavioral measures from A. Working memory task, B. Social cognition task, and C. Language task. 2) The word clouds at the bottom provide a qualitative representation of the correlations for A. Working Memory task, B. Social Cognition task, and C. Language task. The size of the word represents correlation strength, and the color represents behavioral category. Note that the font color in the word clouds matches the category colors defined for the boxplots. WM – Working Memory, (T) – Target trials, (NT) – Non-Target trials.*

#

# Supplemental results SR1: Brain visualization of top region-level accuracies

In the working memory task, some of the strongest discriminators were connections within and between the lateral occipital complex and the temporoparietal regions, likely facilitating working memory and attentional mechanisms (Barch et al., 2013; Igelström & Graziano, 2017) to recognize and remember visually presented stimuli (Supplementary Figure SF2A). Some of these regions also showed relatively high levels of integration across the brain (based on column-wise average in the 148x148 matrix), indicated by the yellowish regions in Supplementary Figure SF3A. Additionally, the integration was slightly higher around the supplementary motor areas, possibly supporting the culmination of all the cognitive processing into verbal responses provided by participants in each trial. In addition to the visual regions, some of the strongest discriminatory connections in the social cognition task were associated with temporal, medial prefrontal, and supplementary motor regions (see Supplementary Figure SF2B), previously implicated in social cognition, self-referential processing, and processing animations (Barch et al., 2013; Schurz et al., 2014). Together, these connections likely facilitated the underlying cognition required to infer the social intentions of moving shapes. This is further supported by higher-than-average region level accuracies (based on column-wise average in 148x148 matrix) in the visual, medial prefrontal, temporal and temporoparietal regions (see Supplementary Figure SF3B). In the language task, some of the top connections were found within and between the temporal areas associated with auditory and language processing and other high-level cognitive areas including frontal gyri and anterior cingulate regions (see Supplementary Figure SF2C). These regions typically support high-level semantic cognition involved in processing language and making inferences about stories (Barch et al., 2013). Similarly, the average region level accuracies were higher for the somatomotor areas, indicating their central role in this task, as shown in Supplementary Figure SF3C. The most notable connections in the motor task encompassed the motor and visual areas, along with some connections associated with the inferior frontal areas (Supplementary Figure SF2D). This is also reflected by the average region level accuracies (see Supplementary Figure SF3D).

*Figure SF2: Top 2% of the cortical region-to-region connections (in terms of accuracy) that showed successful discrimination between task and rest based on dynamic functional connectivity are mapped on the brain surface using the visualization tool, BrainNet Viewer (Xia et al., 2013), for the four successful tasks - A. Working memory task, B. Social cognition task, C. Language task, and D. Motor task. The nodes represent brain regions, and the edges represent the interactions between these regions that resulted in successful classification with accuracy values among the top 2%.*

*Figure SF3: Brain surface renderings of average prediction accuracy based on dynamic functional connectivity across all connections associated with each individual brain region, for A. Working memory task, B. Social cognition task, C. Language task, and D. Motor task. For each region, the mean of its column in the 148x148 region-level accuracy matrix was computed, resulting in an average accuracy value for the region. This accuracy value associated with a region determines the region’s color.*

# Supplemental results SR2: Relationship between classification weights and task experimental design

Since mean task activation effects were regressed out prior to estimating dynamic FC and applying logistic regression classification, we did not expect the classification weights to resemble the average task design for any of the tasks. As shown in Supplementary Figure SF4, classification weights were indeed decoupled from task blocks. Moreover, we calculated correlations between the average weight vector of above-threshold connections and the group average of each HRF-convolved task regressor separately to quantitatively explore any relationships. However, we found no statistically significant correlations (p > 0.5 uncorrected). This indicates that on average, the discrimination between task and rest may have been driven by differences in dynamic functional connectivity, thus unlikely representing trivial stimuli-driven distinctions. Further investigations into this relationship at the level of time points (weights) and individuals (task regressors) may offer deeper insights. However, this is currently beyond the scope of our work.

**

*Figure SF4:Hemodynamic response function (HRF) - convolved task design (red trace), averaged across individuals, and the classification weights, averaged across all region-to-region connections that successfully discriminated (accuracies exceeding the set threshold) between dynamic functional connectivity associated with task and rest (blue trace) after the removal of effects due to task design, head motion and physiological noise, for the four successful tasks, i.e., A. Working memory task, B. Social cognition task, C. Language task, and D. Motor task. There appears to be no explicit relationship between the block structure of task conditions and the feature weights determining classification performance.*

*Figure SF5: Accuracy of classifying between tasks and rest using dynamic functional connectivity at the brain region level, after removal of task activation, head motion and physiological effects. Matrices of prediction accuracies (148×148) exceeding the pre-defined threshold, prior to down sampling to canonical networks for A. Relational task, B. Gambling task, and C. Emotion task. Regions are delineated based on the Craddock volumetric atlas, with two regions of the brain stem removed. They are grouped according to canonical networks demarcated by magenta boundaries and indicated by labels. Each yellow square within a matrix represents a region-to-region connection whose prediction accuracy exceeded the set threshold.* *Matrices for the other 4 tasks have been included in the main text, left panel of Figure 2.*

*Figure SF6: Matrices of accuracies (148x148) of classification between tasks and rest based on time-averaged (static) functional connectivity, after removal of task activation, head motion and physiological effects and prior to down sampling to canonical networks for A. Working memory task B. Social cognition task, C. Language task, D. Motor task, E. Relational task, F. Gambling task, and G. Emotion task. Regions are delineated based on the Craddock volumetric atlas, with two regions of the brain stem removed. They are grouped according to canonical networks demarcated by blue boundaries and indicated by labels.*

*Figure SF7: Histogram representing the distribution of accuracies from classifying between task and rest based on static functional connectivity, for A. Working memory task, B. Social cognition task, C. Language task, D. Motor task, E. Relational task, F. Gambling task, and G. Emotion task. Each magenta bin represents the total number of region-to-region connections associated with a range of accuracies. For every task, the accuracies are largely concentrated around the chance-level value of 50%.*

*Figure SF8: Scatter plots with confidence intervals showing the associations between notable behavioral measures (y-axes) and task identifiability measures (x-axes) in the three HCP tasks, i.e., A. Working memory task, B. Social cognition task, and C. Language task. These associations have been controlled for age and gender. The title of each plot represents the category of the corresponding behavioral measure. Correlation values (r) and the Bonferroni corrected p-values of significance (p<0.05/192) for all the scatter plots have been shown in boxes at the right for each task.*

# Supplemental Analysis SA1: MTPA using minimally pre-processed data - prior to removal of head motion, physiological and task activation effects

To further explore the effect of head motion, physiological sources, and average task activation on the extent of discrimination possible between the 7 tasks and rest based on DFC, we repeated the same analysis prior to the additional pre-processing step. DFC was estimated following the same procedure as described in section 2.2 of the main text. Similarly, the same MTPA pipeline, as described in section 2.4 of the main text, was followed throughout, including training classification models for 11,175 distinct region-to-region connections using 10-fold cross-validation, followed by summarizing the connections into large-scale brain network interactions (Yeo et al 2011).

Following the same guidelines described in the original analysis, 70% was set as the accuracy threshold here since it enabled a reasonable balance between sensitivity and specificity. Using a lower threshold of 60% provided low specificity with more than 65% of the network connections exceeding the threshold on average across all 7 tasks, whereas a higher threshold of 80% provided low sensitivity with fewer than 1% of connections exceeding the threshold on average. All accuracies reported here were also estimated out of sample. The classification accuracies estimated in the training set using 10-fold cross-validation were comparable, indicating successful pattern classification without model overfitting. Note that 2 out of the 150 regions largely overlapped with the brain stem and hence were removed from analysis.

Supplementary Figure SF9 shows the 148×148 matrix of out-of-sample classification accuracies prior to summarizing (i.e., down sampling) in terms of the 20 established canonical networks for all the 7 tasks. Each network connection in isolation was capable of an accuracy exceeding 70%. Possible interactions between multiple network connections were not considered. Supplementary Figure SF10 shows a circular graph representation of the summarized 20×20 matrix of averaged out-of-sample classification accuracies for each of the tasks for which accuracy exceeded the threshold, i.e., 5 tasks.

*Figure SF9: Accuracy of classifying between minimally pre-processed tasks and rest using dynamic functional connectivity at the brain region level. Matrices of prediction accuracies (148×148) exceeding the pre-defined threshold, prior to down sampling to canonical networks for A. Working memory task, B. Social cognition task, C. Language task, D. Motor task, E. Relational task, F. Gambling task, and G. Emotion task. Regions are delineated based on the Craddock volumetric atlas, with two regions of the brain stem removed. They are grouped according to canonical networks demarcated by magenta boundaries and indicated by labels. Each yellow square within a matrix represents a region-to-region connection whose prediction accuracy exceeded the set threshold.*


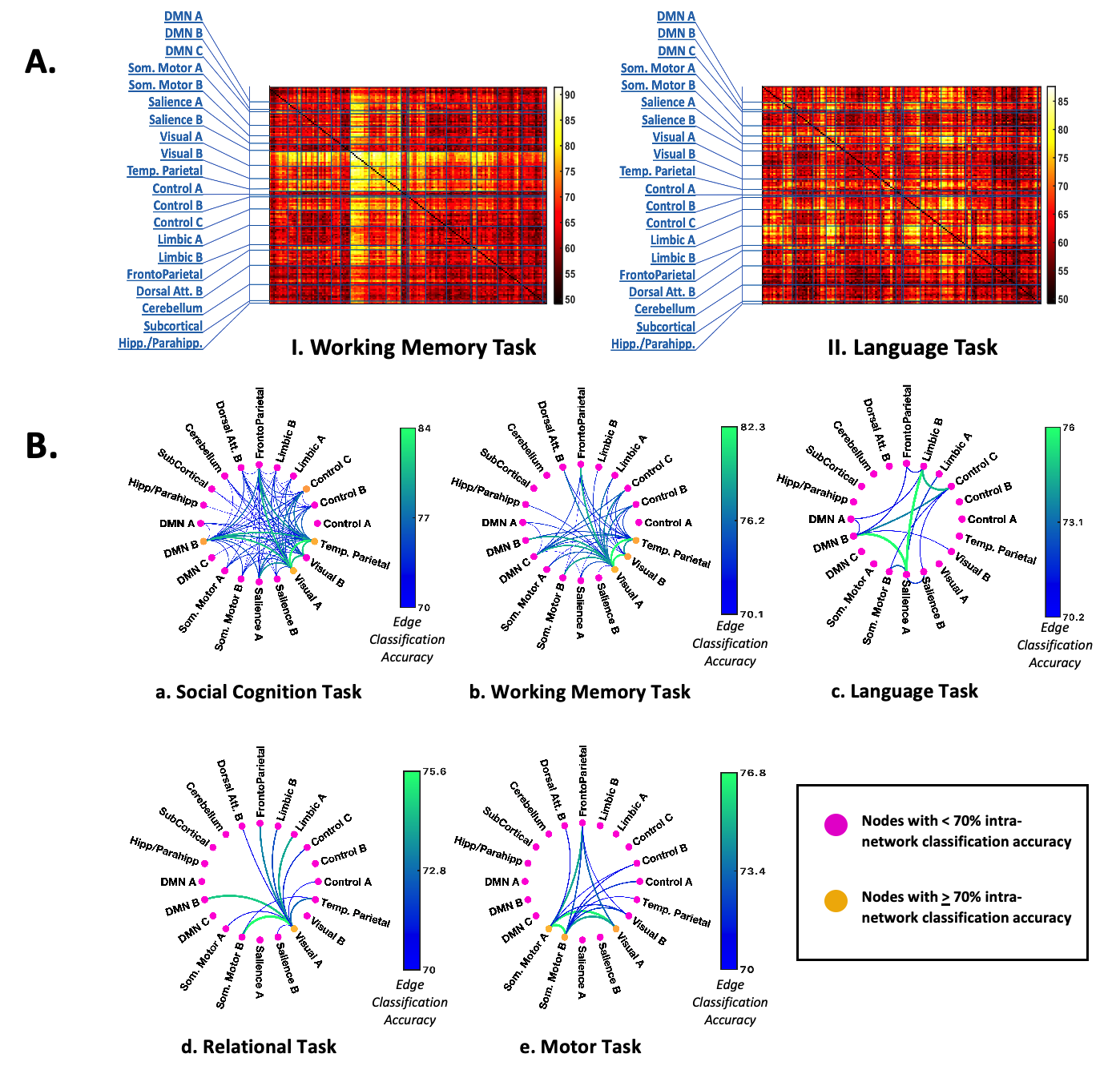


*Figure SF10: Accuracy of classifying between minimally pre-processed tasks and rest using dynamic functional connectivity at the brain network level. Circular graph representations (using the toolbox by Kassebaum (2020)) of inter- and intra-network connections yielding average prediction accuracies exceeding the pre-defined threshold for five tasks, i.e., a. Social Cognition task, b. Working Memory task, c. Language task, d. Relational task, and e. Motor task. Each node represents an intra-network connection while each edge represents an inter-network connection. The edge weights and edge colors (indicated by color bars) represent out-of-sample classification accuracy. Yellow nodes represent intra-network connections whose dynamics enable prediction with accuracy exceeding 70%, while pink nodes represent intra-network connections whose dynamics enable weaker prediction at below-threshold accuracies.*

## Network configurations enabling successful discrimination between task and rest based on dynamic functional connectivity

As expected, we found several additional connections compared to the residual connectivity analysis in the main manuscript. Importantly, apart from the four tasks in the main analysis, the relational task also showed successful network connections exceeding the threshold. Overall, the prediction accuracies were much higher compared to the main analysis. This is understandable given the retention of mean task activation effect among other confounds in this supplementary analysis.

There were many more overlapping intra- and inter-network connections across most tasks. For example, inter-network connections of the low-level visual A network were prominent in all the tasks except the language task. The functionally versatile DMN B sub-network also participated in multiple tasks. Consistent with the frontoparietal network’s recognized role in attentional allocation and memory processes (Naghavi & Nyberg, 2005), its dynamics were strongly modulated by every task. Similarly, salience network connections may have supported attentional reallocation to salient stimuli and higher cognitive processing (Menon & Uddin, 2010; Seeley et al., 2007) across tasks through dynamic alterations. Inter-network dynamics of the limbic network, mainly comprising frontal and temporal regions, may have contributed to higher cognitive processing such as learning, emotion, executive functions, etc. (Dixon et al., 2017; Jackson et al., 2018) crucial to successfully performing a cognitive task. Finally, connections belonging to the frontotemporal network dominated control C network could be explained by the network’s recognized role in executive task control (Dosenbach et al., 2007; Seeley et al., 2007) pivotal for task engagement.

Some of the connections appeared to be performing specialized roles for each task. For instance, the temporoparietal network shared connections with 95% of the brain networks in the social cognition task and with 65% of them in the working memory task. Additionally, in the working memory task, we also observed notable participation by DMN C sub-network, particularly by sharing an edge with visual A (78.8% accuracy), possibly supporting visual memory and contextual processing (Aminoff et al., 2013; Garoff et al., 2005). Inter-network connectivity dynamics of hippocampal regions, widely known for their role in memory processing and working memory load (Rissman et al., 2008; Treves & Rolls, 1994; Yonelinas et al., 2001), were also found crucial to distinguish task and rest specifically in working memory and social cognition tasks. Specifically, In the language task, some of the most notable network connections are known for higher cognitive processing and task execution, such as DMN B, salience, limbic and control C. For example, the highest accuracy connection was between salience A and DMN B (76% accuracy) followed by salience A and limbic B (75.9% accuracy), control C and limbic B (74.6% accuracy) and control C and DMN B (73.5%). Similarly, some of the top connections observed in the relational task were between the low-level visual A network and high-level networks such as DMN B, limbic A and frontoparietal. Particularly notable connections were between visual A and DMN B (74.5% accuracy), visual A and limbic A (73.9% accuracy), and visual A and frontoparietal (73.2% accuracy) networks. These connections, along with the intra-network connection of visual A (72.7% accuracy), would have been necessary for the visually intense relational pattern task. Finally, the motor task could be discriminated from rest most accurately based on dynamics of the primary and higher-level somatomotor networks, as expected. Specifically, some of the top connections were between the primary and higher-level somatomotor networks A and B (76.8% accuracy), somatomotor A and visual A (76.4% accuracy) and somatomotor A and frontoparietal (74.8% accuracy). Since this task largely involved movement, the within-network accuracy of the primary somatomotor network A (76.1%) was also above threshold.

## Classifying task and rest using static functional connectivity

To compare the discriminability between task and rest enabled by minimally pre-processed dynamic and static functional connectivity measures, we followed the same procedure, as described in section 2.3 and 2.4.2 of the main text, to calculate MTPA accuracies for static functional connectivity. As expected, most network connections were concentrated around 50% prediction accuracy with none exceeding the 70% mark. We compared the absolute average prediction accuracies obtained from dynamic and static functional connectivity by averaging across the whole 20×20 matrix of each task (Supplementary Figure SF11). We found that the average discrimination by static functional connectivity was comparable to chance level (50%) in every task, with a narrow standard deviation. This is consistent with our main finding and previous studies (Cole et al., 2014; Fair et al., 2007). Additionally, as expected, the accuracies from dynamic FC here were higher for every task compared to those calculated in the main analysis, after task activation removal.

*Figure SF11: Average accuracy of classifying task from rest based on time-averaged (blue) and dynamic (red) functional connectivity, with intact head motion confound, physiological confound and mean task activation. Accuracy is shown for each of 7 tasks. The standard deviation across connections is indicated by the vertical line along the top of each bar.*

## Impact of behavior on classifying task and rest using functional connectivity dynamics

Having found that task and rest can be accurately classified for five of the 7 tasks when additional pre-processing steps were omitted, we next sought to investigate whether classification accuracy was impacted by behavior, personality, and other individual-specific measures. We followed the same approach as described in section 2.5. We computed the task identifiability measure for each individual and each task by averaging individual prediction probability estimates across all the region-to-region connections included in the thresholded network configuration. We selected 199 HCP measures belonging to broad categories such as in-scanner task performance, cognition, emotion, personality, etc. Note that there are 199 measures here because of including the relational task metrics since it produced successful network connections in this analysis. The Pearson partial correlation coefficient was used to test whether interindividual variation in each measure associated with classification performance, while controlling for the effects of age and sex. The Bonferroni correction was applied to correct for multiple comparisons across the 199 tests for each task.

For each of the five tasks, we found a significant relationship between the task identifiability measure and several behavioral measures associated with 6 distinct behavioral categories, namely in-scanner task performance, cognition, emotion, personality, alertness, and motor ability. Figure 4 shows boxplots for the significant correlations between classification accuracy and behaviors (p<0.05/199) with an effect size of |r| > 0.1. The boxplots are color coded by the category of the behavioral measure and the word clouds at the right provide a qualitative representation of the box plots. The complete summary of 192 of the measures used here can be found in Supplementary Table ST2. The additional measures corresponded to the task performance metrics of the relational task.

*Figure SF12: 1) Boxplots showing statistically significant correlations (p < 0.05, |r| > 0.1, Bonferroni corrected), with 75% confidence interval, between individual task identifiability measures and behavioral measures from A. Social cognition task, and B. Relational task, after controlling for the influence of age and gender. The remaining boxplots have been shown in a separate figure below, Supplementary Figure SF13. 2) The corresponding word clouds from A. Working memory task, B. Social cognition task, C. Language task, D. Relational task, and E. Motor task, provide a qualitative representation of the correlation, where the size of the word represents correlation strength, and the color represents behavioral category. Note that the font color in the word clouds matches the category colors defined for the boxplots. WM – Working Memory, (T) – Target trials, (NT) – Non-Target trials.*

**

*Figure SF13: Boxplots showing statistically significant correlations (p < 0.05, |r| > 0.1, Bonferroni corrected), with 75% confidence interval, between individual task identifiability measures and behavioral measures for the remaining tasks, i.e., a. Working memory task, b. Language task, and c. Motor task, after controlling for the influence of age and gender. Note that the boxplots for the other two tasks have been shown in Supplementary Figure SF12 (above). WM – Working Memory, (T) – Target trials, (NT) – Non-Target trials.*

As expected, the number of surviving behavioural measures were substantially higher compared to the original analysis, where the effects of head motion, physiological sources and task activation were regressed out. Moreover, we found surviving behavioural measures in two additional tasks, i.e., relational and motor. Consistent with findings from our main analysis, we found significant associations between task identifiability and task-specific in-scanner accuracy measures. Similarly, the task identifiability measure of each task significantly correlated with working memory task measures such as accuracy, reaction times, etc. We also found several significantly correlated measures associated with cognition, including fluid and crystallized cognition, spatial processing ability, executive function, language & reading ability, etc. The association of alertness with the social cognition task identifiability measure was replicated here too.

In addition to emotion, we also found that personality measures exerted a significant influence on the success of discriminating between the connectivity dynamics of task and rest. Perceived stress, emotion recognition and anger were among the emotion measures that showed the strongest associations with the classification performance in most tasks. We also found some other additional emotion measures, such as sadness in the social cognition task and hostility in the relational task. Among the personality measures, we found significant associations in two tasks: between neuroticism and social cognition task, and openness and language task. We also found endurance to be associated with multiple tasks.

## Conclusion

By applying MTPA on minimally pre-processed HCP data, we found several additional connections and associated behaviours (personality traits, etc.) on top of the ones unravelled using residual connectivity estimates. Moreover, successful network connections were produced in one more task (relational) while surviving behaviours were produced in two additional tasks (relational and motor). Comparison with static functional connectivity showed findings that were consistent with our main analysis. Although the source of these additional connections and behaviors can be attributed to task-related processing during the task condition blocks, there is also evidence for artifactual contamination of FC calculations by head motion and physiological signals, which can potentially influence the classification performance (Power et al., 2012). Furthermore, these artifacts are also known to impact individual differences in behavior and their associated correlations (Bolton et al., 2020; Siegel et al., 2017). Therefore, causal inferences linking these additional results with actual task-related effects need to be considered with caution.

However, it is worth noting that the influence of these confounds can be considered detrimental or diagnostic based on the overall aim of such multivariate analysis. The goal of standard univariate analyses is generally limited to identifying brain regions associated with cognitive states at the group level. However, with multivariate techniques like MTPA, the purpose can also be purely predictive, i.e., to distinguish and decode distinct cognitive states and identify the connections and features most suitable to achieve this. Therefore, any additional sources of information (apart from neural BOLD signals) including head motion and physiological signals would thus be crucial since they would contribute to the overall predictive capability. Following the predictive pathway, MTPA can be flexibly adopted in the future to investigate the relative sensitivity of functional connections to neural signals and non-neural confounds, in terms of their dynamics in specific contexts. Similarly, individual-specific behaviors most susceptible to neural and non-neural dynamics can be delineated, and cautiously considered while recruiting for future task paradigms. Furthermore, the predictive pathway of MTPA could also lead to the development of diagnostic capabilities in neuropsychiatry research.

*Table ST2: Intuitive names and assessments associated with all the 199 HCP measures included in the study.*

| Category | HCP Formal Name | Intuitive Name | Assessment |
| --- | --- | --- | --- |
| Age | Age_in_Yrs | Age | Demographics assessment |
| Sex | Gender | Gender |  |
| Zygosity | ZygositySR | Zygosity |  |
| Task Performance | WM_Task_Acc | Working Memory accuracy | In-Scanner Task Performance |
|  | WM_Task_Median_RT | Working Memory reaction time |  |
|  | WM_Task_2bk_Acc | Working Memory 2bk accuracy |  |
|  | WM_Task_2bk_Median_RT | Working Memory 2bk reaction time |  |
|  | WM_Task_0bk_Acc | Working Memory 0bk accuracy |  |
|  | WM_Task_0bk_Median_RT | Working Memory 0bk reaction time |  |
|  | WM_Task_0bk_Body_Acc | Working Memory 0bk Body accuracy |  |
|  | WM_Task_0bk_Body_Acc_T | Working Memory 0bk Body accuracy (target trials) |  |
|  | WM_Task_0bk_Body_Acc_NT | Working Memory 0bk Body accuracy (nontarget trials) |  |
|  | WM_Task_0bk_Face_Acc | Working Memory 0bk Face accuracy |  |
|  | WM_Task_0bk_Face_Acc_T | Working Memory 0bk Face accuracy (target trials) |  |
|  | WM_Task_0bk_Face_Acc_NT | Working Memory 0bk Face accuracy (nontarget trials) |  |
|  | WM_Task_0bk_Place_Acc | Working Memory 0bk Place accuracy |  |
|  | WM_Task_0bk_Place_Acc_T | Working Memory 0bk Place accuracy (target trials) |  |
|  | WM_Task_0bk_Place_Acc_NT | Working Memory 0bk Place accuracy (nontarget trials) |  |
|  | WM_Task_0bk_Tool_Acc | Working Memory 0bk Tool accuracy |  |
|  | WM_Task_0bk_Tool_Acc_T | Working Memory 0bk Tool accuracy (target trials) |  |
|  | WM_Task_0bk_Tool_Acc_NT | Working Memory 0bk Tool accuracy (nontarget trials) |  |
|  | WM_Task_2bk_Body_Acc | Working Memory 2bk Body accuracy |  |
|  | WM_Task_2bk_Body_Acc_T | Working Memory 2bk Body accuracy (target trials) |  |
|  | WM_Task_2bk_Body_Acc_NT | Working Memory 2bk Body accuracy (nontarget trials) |  |
|  | WM_Task_2bk_Face_Acc | Working Memory 2bk Face accuracy |  |
|  | WM_Task_2bk_Face_Acc_T | Working Memory 2bk Face accuracy (target trials) |  |
|  | WM_Task_2bk_Face_Acc_NT | Working Memory 2bk Face accuracy (nontarget trials) |  |
|  | WM_Task_2bk_Place_Acc | Working Memory 2bk Place accuracy |  |
|  | WM_Task_2bk_Place_Acc_T | Working Memory 2bk Place accuracy (target trials) |  |
|  | WM_Task_2bk_Place_Acc_NT | Working Memory 2bk Place accuracy (nontarget trials) |  |
|  | WM_Task_2bk_Tool_Acc | Working Memory 2bk Tool accuracy |  |
|  | WM_Task_2bk_Tool_Acc_T | Working Memory 2bk Tool accuracy (target trials) |  |
|  | WM_Task_2bk_Tool_Acc_NT | Working Memory 2bk Tool accuracy (nontarget trials) |  |
|  | WM_Task_0bk_Body_Median_RT | Working Memory 0bk Body reaction time |  |
|  | WM_Task_0bk_Body_Median_RT_T | Working Memory 0bk Body reaction time (target trials) |  |
|  | WM_Task_0bk_Body_Median_RT_NT | Working Memory 0bk Body reaction time (nontarget trials) |  |
|  | WM_Task_0bk_Face_Median_RT | Working Memory 0bk Face reaction time |  |
|  | WM_Task_0bk_Face_Median_RT_T | Working Memory 0bk Face reaction time (target trials) |  |
|  | WM_Task_0bk_Face_Median_RT_NT | Working Memory 0bk Face reaction time (nontarget trials) |  |
|  | WM_Task_0bk_Place_Median_RT | Working Memory 0bk Place reaction time |  |
|  | WM_Task_0bk_Place_Median_RT_T | Working Memory 0bk Place reaction time (target trials) |  |
|  | WM_Task_0bk_Place_Median_RT_NT | Working Memory 0bk Place reaction time (nontarget trials) |  |
|  | WM_Task_0bk_Tool_Median_RT | Working Memory 0bk Tool reaction time |  |
|  | WM_Task_0bk_Tool_Median_RT_T | Working Memory 0bk Tool reaction time (target trials) |  |
|  | WM_Task_0bk_Tool_Median_RT_NT | Working Memory 0bk Tool reaction time (nontarget trials) |  |
|  | WM_Task_2bk_Body_Median_RT | Working Memory 2bk Body reaction time |  |
|  | WM_Task_2bk_Body_Median_RT_T | Working Memory 2bk Body reaction time (target trials) |  |
|  | WM_Task_2bk_Body_Median_RT_NT | Working Memory 2bk Body reaction time (nontarget trials) |  |
|  | WM_Task_2bk_Face_Median_RT | Working Memory 2bk Face reaction time |  |
|  | WM_Task_2bk_Face_Median_RT_T | Working Memory 2bk Face reaction time (target trials) |  |
|  | WM_Task_2bk_Face_Median_RT_NT | Working Memory 2bk Face reaction time (nontarget trials) |  |
|  | WM_Task_2bk_Place_Median_RT | Working Memory 2bk Place reaction time |  |
|  | WM_Task_2bk_Place_Median_RT_T | Working Memory 2bk Place reaction time (target trials) |  |
|  | WM_Task_2bk_Place_Median_RT_NT | Working Memory 2bk Place reaction time (nontarget trials) |  |
|  | WM_Task_2bk_Tool_Median_RT | Working Memory 2bk Tool reaction time |  |
|  | WM_Task_2bk_Tool_Median_RT_T | Working Memory 2bk Tool reaction time (target trials) |  |
|  | WM_Task_2bk_Tool_Median_RT_NT | Working Memory 2bk Tool reaction time (nontarget trials) |  |
|  | Social_Task_Perc_Random | Social Cognition random rating |  |
|  | Social_Task_Perc_TOM | Social Cognition rating |  |
|  | Social_Task_Perc_Unsure | Social Cognition unsureness |  |
|  | Social_Task_Perc_NLR | Social Cognition no response |  |
|  | Social_Task_Median_RT_Random | Social Cognition random reaction time |  |
|  | Social_Task_Median_RT_TOM | Social Cognition reaction time |  |
|  | Social_Task_Median_RT_Unsure | Social Cognition unsure reaction time |  |
|  | Social_Task_Random_Perc_Random | Social Cognition random accuracy |  |
|  | Social_Task_Random_Median_RT_Random | Social Cognition reaction time (accurate random) |  |
|  | Social_Task_Random_Perc_TOM | Social Cognition random inaccuracy |  |
|  | Social_Task_Random_Median_RT_TOM | Social Cognition reaction time (inaccurate random) |  |
|  | Social_Task_Random_Perc_Unsure | Social Cognition random unsureness |  |
|  | Social_Task_Random_Median_RT_Unsure | Social Cognition reaction time (random unsureness) |  |
|  | Social_Task_Random_Perc_NLR | Social Cognition no response (random) |  |
|  | Social_Task_TOM_Perc_Random | Social Cognition inaccuracy |  |
|  | Social_Task_TOM_Median_RT_Random | Social Cognition reaction time (inaccurate social) |  |
|  | Social_Task_TOM_Perc_TOM | Social Cognition rating |  |
|  | Social_Task_TOM_Median_RT_TOM | Social Cognition accuracy |  |
|  | Social_Task_TOM_Perc_Unsure | Social Cognition social unsureness |  |
|  | Social_Task_TOM_Median_RT_Unsure | Social Cognition reaction time (social unsureness) |  |
|  | Social_Task_TOM_Perc_NLR | Social Cognition no response (social) |  |
|  | Language_Task_Acc | Language accuracy |  |
|  | Language_Task_Median_RT | Language reaction time |  |
|  | Language_Task_Story_Acc | Language Story accuracy |  |
|  | Language_Task_Story_Median_RT | Language Story reaction time |  |
|  | Language_Task_Story_Avg_Difficulty_Level | Language Story Difficulty |  |
|  | Language_Task_Math_Acc | Language Math accuracy |  |
|  | Language_Task_Math_Median_RT | Language Math reaction time |  |
|  | Language_Task_Math_Avg_Difficulty_Level | Language Math Difficulty |  |
| Motor Ability | Endurance_Unadj | Endurance | NIH Toolbox 2-minute Walk Endurance Test |
|  | Endurance_AgeAdj |  |  |
|  | GaitSpeed_Comp | Gait Speed | NIH Toolbox 4-Meter Walk Gait Speed Test |
|  | Dexterity_Unadj | Manual Dexterity | NIH Toolbox 9-hole Pegboard Dexterity Test |
|  | Dexterity_AgeAdj |  |  |
|  | Strength_Unadj | Strength | NIH Toolbox Grip Strength Test |
|  | Strength_AgeAdj |  |  |
| Cognition | PicSeq_Unadj | Episodic Memory | NIH Toolbox Picture Sequence Memory Test |
|  | PicSeq_AgeAdj |  |  |
|  | CardSort_Unadj | Cognitive Flexibility | NIH Toolbox Dimensional Change Card Sort Test |
|  | CardSort_AgeAdj |  |  |
|  | Flanker_Unadj | Executive Function | NIH Toolbox Flanker Inhibitory Control and Attention Test |
|  | Flanker_AgeAdj |  |  |
|  | PMAT24_A_CR | Fluid Intelligence accuracy | Penn Progressive Matrices |
|  | PMAT24_A_SI | Fluid Intelligence inaccuracy |  |
|  | PMAT24_A_RTCR | Fluid Intelligence reaction time |  |
|  | ReadEng_Unadj | Language & Reading | NIH Toolbox Oral Reading Recognition Test |
|  | ReadEng_AgeAdj |  |  |
|  | PicVocab_Unadj | Language & Vocabulary | NIH Toolbox Picture Vocabulary Test |
|  | PicVocab_AgeAdj |  |  |
|  | ProcSpeed_Unadj | Cognition Speed | NIH Toolbox Pattern Comparison Processing Speed Test |
|  | ProcSpeed_AgeAdj |  |  |
|  | VSPLOT_TC | Spatial Processing accuracy | Variable Short Penn Line Orientation Test |
|  | VSPLOT_CRTE | Spatial Processing reaction time |  |
|  | VSPLOT_OFF | Spatial Processing inaccuracy |  |
|  | SCPT_TP | Sustained Attention true positives | Short Penn Continuous Performance Test |
|  | SCPT_TN | Sustained Attention true negatives |  |
|  | SCPT_FP | Sustained Attention false positives |  |
|  | SCPT_FN | Sustained Attention false negatives |  |
|  | SCPT_TPRT | Sustained Attention reaction time (true positives) |  |
|  | SCPT_SEN | Sustained Attention sensitivity |  |
|  | SCPT_SPEC | Sustained Attention specificity |  |
|  | SCPT_LRNR | Sustained Attention non-response |  |
|  | IWRD_TOT | Verbal Episodic Memory | Penn Word Memory Test |
|  | IWRD_RTC | Verbal Episodic Memory reaction time |  |
|  | ListSort_Unadj | Working Memory | NIH Toolbox List Sorting Working Memory Test |
|  | ListSort_AgeAdj |  |  |
|  | CogFluidComp_Unadj | Fluid Cognition | NIH Toolbox Cognition Fluid Composite |
|  | CogFluidComp_AgeAdj |  |  |
|  | CogEarlyComp_Unadj | Early Childhood Cognition | NIH Toolbox Cognition Early Childhood Composite |
|  | CogEarlyComp_AgeAdj |  |  |
|  | CogTotalComp_Unadj | Total Cognition | NIH Toolbox Cognition Total Composite |
|  | CogTotalComp_AgeAdj |  |  |
|  | CogCrystalComp_Unadj | Crystallized Cognition | NIH Toolbox Cognition Crystallized Composite |
|  | CogCrystalComp_AgeAdj |  |  |
| Emotion | ER40_CR | Emotion Recognition accuracy | Penn Emotion Recognition Test |
|  | ER40_CRT | Emotion Recognition reaction time |  |
|  | ER40ANG | Anger Identification |  |
|  | ER40FEAR | Fear Identification |  |
|  | ER40HAP | Happy Identification |  |
|  | ER4FNOE | Neutral Identification |  |
|  | ER40SAD | Sad Identification |  |
|  | AngAffect_Unadj | Anger | NIH Toolbox Anger-Affect Survey |
|  | AngHostil_Unadj | Hostility | NIH Toolbox Anger-Hostility Survey |
|  | AngAggr_Unadj | Aggression | NIH Toolbox Anger-Aggression Survey |
|  | FearAffect_Unadj | Fear | NIH Toolbox Fear-Affect Survey |
|  | FearSomat_Unadj | Autonomic Fear Arousal | NIH Toolbox Fear-Somatic Arousal Survey |
|  | Sadness_Unadj | Sadness | NIH Toolbox Sadness Survey |
|  | LifeSatisf_Unadj | General Life Satisfaction | NIH Toolbox General Life Satisfaction Survey |
|  | MeanPurp_Unadj | Meaning and Purpose | NIH Toolbox Meaning and Purpose Survey |
|  | PosAffect_Unadj | Positive Affect | NIH Toolbox Positive Affect Survey |
|  | Friendship_Unadj | Perception of Friendship | NIH Toolbox Friendship Survey |
|  | Loneliness_Unadj | Loneliness | NIH Toolbox Loneliness Survey |
|  | PercHostil_Unadj | Perceived Hostility | NIH Toolbox Perceived Hostility Survey |
|  | PercReject_Unadj | Perceived Rejection | NIH Toolbox Perceived Rejection Survey |
|  | EmotSupp_Unadj | Emotional Support | NIH Toolbox Emotional Support Survey |
|  | InstruSupp_Unadj | Instrumental Support | NIH Toolbox Instrumental Support Survey |
|  | PercStress_Unadj | Perceived Stress | NIH Toolbox Perceived Stress Survey |
|  | SelfEff_Unadj | General Self-Efficacy | NIH Toolbox Self-Efficacy Survey |
| Personality | NEOFAC_A | Agreeableness | NEO-FFI Agreeableness |
|  | NEOFAC_O | Openness | NEO-FFI Openness |
|  | NEOFAC_C | Conscientiousness | NEO-FFI Conscientiousness |
|  | NEOFAC_N | Neuroticism | NEO-FFI Neuroticism |
|  | NEOFAC_E | Extraversion | NEO-FFI Extraversion |
| Alertness | MMSE_Score | Cognitive Status | Mini Mental Status Exam |
|  | PSQI_Score | Sleep Quality | Pittsburgh Sleep Questionnaire |
|  | PSQI_Comp1 | Subjective Sleep Quality 1 |  |
|  | PSQI_Comp2 | Sleep Latency |  |
|  | PSQI_Comp3 | Subjective Sleep Quality 3 |  |
|  | PSQI_Comp4 | Sleep Duration |  |
|  | PSQI_Comp5 | Sleep Disturbance |  |
|  | PSQI_Comp6 | Use of Sleep Meds |  |
|  | PSQI_Comp7 | Daytime Dysfunction |  |
|  | PSQI_Min2Asleep | Minutes to fall asleep |  |
|  | PSQI_DayStayAwake | Trouble staying awake during day |  |
| Substance Use | Total_Drinks_7days | Total alcohol in past week | Alcohol Use 7-Day Retrospective |
|  | Num_Days_Drank_7days | Number of alcohol days |  |
|  | Avg_Weekday_Drinks_7days | Alcohol during weekdays |  |
|  | Avg_Weekend_Drinks_7days | Alcohol during weekends |  |
| Health & Family History | FamHist_Scz | Schizophrenia or Psychosis | Family History of Psychiatric and Neurologic Disorders |
|  | FamHist_Dep | Depression |  |
|  | FamHist_BP | Bipolar Disorder |  |
|  | FamHist_Anx | Anxiety |  |
|  | FamHist_DrgAlc | Drug or Alcohol Problems |  |
|  | FamHist_Alz | Alzheimer's Disease |  |
|  | FamHist_PD | Parkinson's Disease |  |
|  | FamHist_TS | Tourette's Syndrome |  |
|  | FamHist_None | No disorders |  |
| Brain Volume | FS_LCort_GM_Vol | Left Cortical Gray Matter volume | FreeSurfer Summary Statistics |
|  | FS_RCort_GM_Vol | Right Cortical Gray Matter volume |  |
|  | FS_TotCort_GM_Vol | Total cortical Gray Matter volume |  |
|  | FS_SubCort_GM_Vol | Subcortical Gray Matter volume |  |
|  | FS_Total_GM_Vol | Total Gray Matter volume |  |
|  | FS_L_WM_Vol | Left White Matter volume |  |
|  | FS_R_WM_Vol | Right White Matter volume |  |
|  | FS_Tot_WM_Vol | Total White Matter volume |  |

# Supplemental References

Aminoff, E. M., Kveraga, K., & Bar, M. (2013). The role of the parahippocampal cortex in cognition. *Trends in Cognitive Sciences, 17*(8), 379-390. doi:<https://doi.org/10.1016/j.tics.2013.06.009>

Barch, D. M., Burgess, G. C., Harms, M. P., Petersen, S. E., Schlaggar, B. L., Corbetta, M., . . . Consortium, W. U.-M. H. (2013). Function in the human connectome: task-fMRI and individual differences in behavior. *Neuroimage, 80*, 169-189. doi:10.1016/j.neuroimage.2013.05.033

Bolton, T. A. W., Kebets, V., Glerean, E., Zöller, D., Li, J., Yeo, B. T. T., . . . Van De Ville, D. (2020). Agito ergo sum: Correlates of spatio-temporal motion characteristics during fMRI. *Neuroimage, 209*, 116433. doi:<https://doi.org/10.1016/j.neuroimage.2019.116433>

Cole, M. W., Bassett, D. S., Power, J. D., Braver, T. S., & Petersen, S. E. (2014). Intrinsic and task-evoked network architectures of the human brain. *Neuron, 83*(1), 238-251. doi:10.1016/j.neuron.2014.05.014

Dixon, M. L., Thiruchselvam, R., Todd, R., & Christoff, K. (2017). Emotion and the prefrontal cortex: An integrative review. *Psychol Bull, 143*(10), 1033-1081. doi:10.1037/bul0000096

Dosenbach, N. U., Fair, D. A., Miezin, F. M., Cohen, A. L., Wenger, K. K., Dosenbach, R. A., . . . Petersen, S. E. (2007). Distinct brain networks for adaptive and stable task control in humans. *Proc Natl Acad Sci U S A, 104*(26), 11073-11078. doi:10.1073/pnas.0704320104

Fair, D. A., Schlaggar, B. L., Cohen, A. L., Miezin, F. M., Dosenbach, N. U., Wenger, K. K., . . . Petersen, S. E. (2007). A method for using blocked and event-related fMRI data to study "resting state" functional connectivity. *Neuroimage, 35*(1), 396-405. doi:10.1016/j.neuroimage.2006.11.051

Fan, R.-E., Chang, K.-W., Hsieh, C.-J., Wang, X.-R., & Lin, C.-J. (2008). LIBLINEAR: A library for large linear classification. *Journal of machine learning research, 9*(Aug), 1871-1874.

Garoff, R. J., Slotnick, S. D., & Schacter, D. L. (2005). The neural origins of specific and general memory: the role of the fusiform cortex. *Neuropsychologia, 43*(6), 847-859. doi:<https://doi.org/10.1016/j.neuropsychologia.2004.09.014>

Igelström, K. M., & Graziano, M. S. A. (2017). The inferior parietal lobule and temporoparietal junction: A network perspective. *Neuropsychologia, 105*, 70-83. doi:<https://doi.org/10.1016/j.neuropsychologia.2017.01.001>

Jackson, R. L., Bajada, C. J., Rice, G. E., Cloutman, L. L., & Lambon Ralph, M. A. (2018). An emergent functional parcellation of the temporal cortex. *Neuroimage, 170*, 385-399. doi:<https://doi.org/10.1016/j.neuroimage.2017.04.024>

Kassebaum, P. (2020). circularGraph: GitHub. Retrieved from <https://github.com/paul-kassebaum-mathworks/circularGraph>

Menon, V., & Uddin, L. Q. (2010). Saliency, switching, attention and control: a network model of insula function. *Brain Struct Funct, 214*(5-6), 655-667. doi:10.1007/s00429-010-0262-0

Naghavi, H. R., & Nyberg, L. (2005). Common fronto-parietal activity in attention, memory, and consciousness: Shared demands on integration? *Consciousness and Cognition, 14*(2), 390-425. doi:<https://doi.org/10.1016/j.concog.2004.10.003>

Power, J. D., Barnes, K. A., Snyder, A. Z., Schlaggar, B. L., & Petersen, S. E. (2012). Spurious but systematic correlations in functional connectivity MRI networks arise from subject motion. *Neuroimage, 59*(3), 2142-2154. doi:10.1016/j.neuroimage.2011.10.018

Rissman, J., Gazzaley, A., & D'Esposito, M. (2008). Dynamic Adjustments in Prefrontal, Hippocampal, and Inferior Temporal Interactions with Increasing Visual Working Memory Load. *Cerebral Cortex, 18*(7), 1618-1629. doi:10.1093/cercor/bhm195

Schurz, M., Radua, J., Aichhorn, M., Richlan, F., & Perner, J. (2014). Fractionating theory of mind: A meta-analysis of functional brain imaging studies. *Neuroscience & Biobehavioral Reviews, 42*, 9-34. doi:<https://doi.org/10.1016/j.neubiorev.2014.01.009>

Seeley, W. W., Menon, V., Schatzberg, A. F., Keller, J., Glover, G. H., Kenna, H., . . . Greicius, M. D. (2007). Dissociable Intrinsic Connectivity Networks for Salience Processing and Executive Control. *The Journal of Neuroscience, 27*(9), 2349-2356. doi:10.1523/jneurosci.5587-06.2007

Siegel, J. S., Mitra, A., Laumann, T. O., Seitzman, B. A., Raichle, M., Corbetta, M., & Snyder, A. Z. (2017). Data Quality Influences Observed Links Between Functional Connectivity and Behavior. *Cerebral Cortex, 27*(9), 4492-4502. doi:10.1093/cercor/bhw253

Treves, A., & Rolls, E. T. (1994). Computational analysis of the role of the hippocampus in memory. *Hippocampus, 4*(3), 374-391. doi:10.1002/hipo.450040319

Xia, M., Wang, J., & He, Y. (2013). BrainNet Viewer: a network visualization tool for human brain connectomics. *PLoS One, 8*(7), e68910. doi:10.1371/journal.pone.0068910

Yonelinas, A. P., Hopfinger, J. B., Buonocore, M. H., Kroll, N. E., & Baynes, K. (2001). Hippocampal, parahippocampal and occipital-temporal contributions to associative and item recognition memory: an fMRI study. *Neuroreport, 12*(2), 359-363. doi:10.1097/00001756-200102120-00035
